# Supplementary material for: Suicide as an incident of severe patient harm: a retrospective cohort study of investigations after suicide in Swedish healthcare in a 13-year perspective
Source: BMJ Open. 2021 Mar 8;11(3):e044068. doi: 10.1136/bmjopen-2020-044068 (PMC7944973; doi:10.1136/bmjopen-2020-044068)
Supplement: Author's manuscript [file bmjopen-2020-044068.draft_revisions.pdf]

BMJ Open is committed to open peer review. As part of this commitment we make the peer review history of every article we publish publicly available.

When an article is published we post the peer reviewers' comments and the authors' responses online. We also post the versions of the paper that were used during peer review. These are the versions that the peer review comments apply to.

The versions of the paper that follow are the versions that were submitted during the peer review process. They are not the versions of record or the final published versions. They should not be cited or distributed as the published version of this manuscript.

BMJ Open is an open access journal and the full, final, typeset and author-corrected version of record of the manuscript is available on our site with no access controls, subscription charges or pay-per-view fees (http://bmjopen.bmj.com).

If you have any questions on BMJ Open's open peer review process please email info.bmjopen@bmj.com

BMJ Open

Suicide as an incident of severe patient harm – a retrospective review of investigations of the healthcare provided to patients prior to their suicide

Journal:	*BMJ Open*
Manuscript ID	bmjopen-2020-044068
Article Type:	Original research
Date Submitted by the Author:	24-Aug-2020
Complete List of Authors:	Fröding, Elin; Jönköping University; Region Jönköpings län<br>Gäre, Boel Andersson; Jönköping University; Region Jönköpings län<br>Westrin, Åsa; Lund University Department of Clinical Sciences Malmo, Division of Psychiatry, Lund University ; Psychiatry Research Skåne, Office for Psychiatry and Habilitation, Region Skåne<br>Ros, Axel; Jönköping University; Region Jönköpings län
Keywords:	Health & safety < HEALTH SERVICES ADMINISTRATION & MANAGEMENT, Quality in health care < HEALTH SERVICES ADMINISTRATION & MANAGEMENT, Risk management < HEALTH SERVICES ADMINISTRATION & MANAGEMENT, Suicide & self-harm < PSYCHIATRY

SCHOLARONE™
Manuscripts

I, the Submitting Author has the right to grant and does grant on behalf of all authors of the Work (as defined in the below author licence), an exclusive licence and/or a non-exclusive licence for contributions from authors who are: i) UK Crown employees; ii) where BMJ has agreed a CC-BY licence shall apply, and/or iii) in accordance with the terms applicable for US Federal Government officers or employees acting as part of their official duties; on a worldwide, perpetual, irrevocable, royalty-free basis to BMJ Publishing Group Ltd ("BMJ") its licensees and where the relevant Journal is co-owned by BMJ to the co-owners of the Journal, to publish the Work in this journal and any other BMJ products and to exploit all rights, as set out in our licence.

The Submitting Author accepts and understands that any supply made under these terms is made by BMJ to the Submitting Author unless you are acting as an employee on behalf of your employer or a postgraduate student of an affiliated institution which is paying any applicable article publishing charge ("APC") for Open Access articles. Where the Submitting Author wishes to make the Work available on an Open Access basis (and intends to pay the relevant APC), the terms of reuse of such Open Access shall be governed by a Creative Commons licence – details of these licences and which Creative Commons licence will apply to this Work are set out in our licence referred to above.

Other than as permitted in any relevant BMJ Author's Self Archiving Policies, I confirm this Work has not been accepted for publication elsewhere, is not being considered for publication elsewhere and does not duplicate material already published. I confirm all authors consent to publication of this Work and authorise the granting of this licence.

For peer review only

Title page

Suicide as an incident of severe patient harm – a retrospective review of investigations of the healthcare provided to patients prior to their suicide

Authors

PhD. Elin Fröding, Prof. Boel Andersson Gäre, Prof. Åsa Westrin, MD. Axel Ros

Corresponding author

Name and address: Elin Fröding, Höglandssjukhuset Hus 34 pl 4, 575 81 Eksjö, Sweden

Mail and phone number: Elin.froding@rjl.se, +4670-3577396

Co-authors

Prof. Boel Andersson Gäre, Jönköping University, Jönköping, Sweden

Prof. Åsa Westrin, Lund University, Lund, Sweden

MD. Axel Ros, Jönköping University, Jönköping, Sweden

Key words

Health & safety, patient safety, risk management, quality in health care, suicide

Word count

3892

For peer review only

ABSTRACT

Objectives

The reporting of suicides among recipients of healthcare services has been mandatory in Sweden since 2006-2017. This study, adopting a 13-year perspective, aimed to explore how this mandatory reporting has influenced associated investigations conducted by the healthcare services, and also examined the lessons obtained, and whether any suicide-prevention-related improvements in terms of patient safety had followed.

Design and settings

This is a retrospective study of reports from Swedish primary and secondary healthcare after suicide to the regulatory authority in Sweden. Three cohorts of suicide cases, each from a different time period, were chosen for analysis. Complete reports of the incident investigations conducted by the healthcare providers with associated patient records and the subsequent decisions of the supervisory authority were analyzed by using a coding scheme.

Results

The investigations largely adopted a microsystem perspective, focusing on final patient contact, throughout the overall study period. Updating existing or developing new routines as well as educational actions had been increasingly proposed, while sharing conclusions across departments was rarely recommended.

Conclusions

The mandatory reporting of suicides as potential cases of patient harm was shown to be restricted to information transfer between healthcare providers and the supervisory authority, rather than fostering participative improvement of patient safety for suicidal patients.

The similarity in outcomes across the cohorts, regardless of changes in legislation, suggests that the investigations were adapted to suit the structure of the authority's reports rather than the specific incident type, and that no new service improvements or lessons are being identified.

To develop more sophisticated infrastructures for investigation, learning, and information-sharing, it is necessary to learn more about preconditions and complexity in the analysis of suicides and the suicidal process.

A shift in investigations' recommendations and reports should be encouraged, to also include learning from successfully treated and resolved suicide-related crises.

Strengths and limitations of the study

- To our knowledge, this is the first evaluation of the outcomes of investigations of specific types of patient harm over time, here exemplified by suicide.
- All investigations concerned the same kind of incident; suicides, and the data were population-based.

For peer review only - http://bmjopen.bmj.com/site/about/guidelines.xhtml

- All data were based on the healthcare providers' investigations and reports to the supervisory authority, the content in these reports is regulated by law; however, the quality of analysis differs, which was not evaluated in this study.
- All data collection and categorization were conducted by only one researcher, which was vulnerable to bias, however; this ensured a high level of consistency.

BACKGROUND

Deaths that occur as a result of patient harm represent a contrast to healthcare services' aim of a high level of patient safety, and such incidents can serve as powerful motivators for learning and improvement.[1 2] In recent decades, efforts to increase patient safety have been intensified. In particular, the reporting and investigating of cases of severe patient injury in order to identify risks and improve patient safety have become widespread safety-improvement strategies.[2] This reflects a Safety-I perspective regarding patient safety, and assumes that adverse outcomes are caused by identifiable failures or malfunctions of specific components different from situations when things go right.[3 4] Similarly, root cause analysis (RCA) has become one of the most widespread tools used in the investigation of healthcare-related incidents, and presumes that such incidents can be explained by linear cause-effect chains.[5 6] Determining what had happened and why an incident occurred should not be the final goal of an incident investigation; the identification of gaps in service provision and means of improving relevant areas of the healthcare organization are important for improving safety.[7] To successfully learn from past incidents, methods to sustainably record and share relevant data are essential.[8 9] However, prior studies have shown that, in healthcare, post-incident investigations usually provide little learning beyond the staff and units involved.[10 11] Thus, the actual value of incident-reporting systems and the RCA approach in healthcare has been questioned.[8 12-15] With the introduction of new concepts in patient safety, such as Safety-II and resilient healthcare, new approaches for improving healthcare have focused on learning from all occurrences in daily practice: to identify both those factors that support a good outcome and those that increase the risk of patient harm.[3 4]

Swedish law states that events with severe patient harm, as well as events involving risk of severe patient harm, that could have been avoided if appropriate actions had been taken by healthcare professionals, should be reported to the supervisory authority.[16] This report to the authority should be preceded by an investigation, conducted by the healthcare providing organization, of the healthcare services provided to the patient before the adverse event. The content of the investigation is regulated by law, and requires identification of the contributory causes of the incident and of service improvements that may prevent the reoccurrence of such an incident.

Suicide is a global health problem with an estimated 800 000 deaths worldwide every year.[17] A large proportion of the individuals who die from suicide have close contact with healthcare professionals in the time before their deaths.[18 19] Further, post-suicide psychological autopsies have found that approximately 90% of suicide victims have psychiatric illnesses at the time of their deaths.[20] This suggests that healthcare professionals play an important role in suicide prevention.[21-23]

In an effort to understand whether failures in any area of the healthcare system have contributed to suicide, and in an attempt to improve suicide-prevention, the Swedish National Board of Health and Welfare in 2006 stipulated that all suicides that occur among patients who were receiving healthcare or were in contact with healthcare services within the four weeks preceding the event must be reported to the authority by the healthcare provider.[24] This remained mandatory regardless of whether the provider determined the suicide to be preventable. In September 2017, this regulation was updated to state that only suicides regarded as "severe patient harm" (i.e., preventable) must be reported to the supervisory authority.[25]

For peer review only - http://bmjopen.bmj.com/site/about/guidelines.xhtml

Before 2011, the supervisory authority performed their own investigations of incidents, and had the power to reprimand the provider and responsible staff. The role of the supervisory authority changed in 2011, when the Swedish Patient Safety Act (2010:659)[16] was implemented. This new law made healthcare organizations responsible for patient-safety improvement, and the role of the supervisory authority was changed to review the investigations made by the providers, and ensure that they were satisfactorily fulfilled and that appropriate actions had been taken to ensure a high level of patient safety. In particular, the authority determines whether the healthcare provider has fulfilled their legislated duties, or whether there are shortcomings in the investigation, in which case the authority may recommend revisions or conduct a site visit to inspect the healthcare provider.

To our knowledge, there are no published evaluations of the outcomes of investigations of specific types of patient harm over time, here exemplified by suicide.

The objective of this study was to explore how mandatory reporting of suicide cases as incidents of potential patient harm has influenced the investigations of healthcare systems. To perform this, a 13-year perspective was adopted, and the lessons and possible improvements for patient safety regarding suicide prevention were examined.

METHODS

Cases

Three cohorts of suicide cases, each from a different time period, that were reported to the supervisory authority were chosen for analysis. Cohort 1 comprised the cases reported to the supervisory authority in 2006, from the time the reporting of suicides became mandatory, to 2007 (n = 279). Cohort 2 comprised all suicides reported in 2015, this represented a period when mandatory reporting was well-established among healthcare providers (n = 436). Cohort 3 comprised all reported suicides from September 1, 2017, which was the time the law regarding reporting was changed, to November 30, 2019 (n = 316).

Complete reports of the incident investigations conducted by the healthcare providers with associated patient records and the subsequent decisions of the supervisory authority were obtained from the supervisory authority. Every individual suicide case was given a code number and the patient's demographic data and treatment received in the months preceding his/her death were registered. Major diagnoses were documented and coded in accordance with the International Statistical Classification of Diseases and related Health Problems, 10th revision (i.e., ICD-10).

Categorization of data

A coding scheme was used to categorize the contributory causes of the respective suicides, the actions reported in the investigations and the decisions of the authority. The same coding scheme was used in a prior study of reported suicide cases in Sweden.[11] This scheme is based on the general categories used in the most common method of investigating adverse events in Swedish healthcare, which is in turn based on RCA.[26] To make the categorization more specific, four of the major categories were divided into additional subcategories. The categories are shown in table 2 and 3. Every category was described and exemplified and a category of "others" was added in case none of the other categories was considered appropriate. In this present study, the contributory causes were reported as "deficiencies." Meanwhile, an "action" was defined as any intervention performed in attempt to prevent new suicides: therefor, actions taken to prevent reported suicides (telephone calls, resuscitations) or actions aimed at informing family members or staff that a suicide had occurred were not registered as actions in this study. Separate notes were made when a deficiency or action was related to a healthcare-service routine, as well as in regard to how learning from the investigation was

For peer review only - http://bmjopen.bmj.com/site/about/guidelines.xhtml

described. To ensure consistency, all data collection and categorization were conducted by only one researcher (EF), a psychiatrist with extensive experience in patient-safety issues.

Organizational levels

Classification of the organizational levels of deficiencies and actions was conducted to better understand where in the organizational system the identified deficiencies and actions were situated. The deficiencies and actions were coded based on a micro-meso-macro-perspective.[27] Microsystems were defined as the basic elements of the healthcare services provided for the patient, such as the inpatient or outpatient care unit. The mesosystem encompassed interactions between different microsystem units, such as cooperation between departments or different healthcare providers. The macrosystem involved the entire healthcare system, such as legislation, political prioritizations, and national policies on healthcare. For each case, the highest organizational level for each deficiency and action was coded.

Supervisory authority

The mandate stipulated to the authority by legislation differed between cohort 1 and cohorts 2 and 3, hence the formulation of the decisions also differed. In this paper, to facilitate comparison among these outcomes, for all cohorts only decisions categorized as “immediate approval” and “inspection” were noted, as these remained unchanged. A note was made if a physician employed by the supervisory authority was involved in the decision-making.

Statistical analyses

Frequencies for each category, organizational hierarchal level of deficiencies and actions, and decisions of the supervisory authority were analyzed per individual and aggregated per cohort.

Chi-square tests of independence were used to compare the number of new routines and the absence of routines within the same cohort, as well as the proportion of the organizational hierarchy of deficiencies and actions between cohorts. We considered a two-sided p value of < 0.05 to indicate statistical significance. As the pre-requisites differed between the cohorts, no further statistical analyses to compare the cohorts were judged to be possible.

The statistical analyses were performed using IBM SPSS Statistics 24.

Ethical review

According to the Swedish *Act Concerning the Ethical Review of Research Involving Humans* (2003:460) and an advisory opinion from the Regional Ethical Review Board (no. 2017/234), this study did not require an ethical review as it did not include human participants.

Patient and Public Involvement

Patients or public were not involved in this study.

RESULTS

Cases

Demographic data for the cases showed similarities across the cohorts, with a dominance of men and a majority of cases reported by psychiatric care. One-fourth of the cases died from suicide within one day of their last contact with a healthcare professional; half of the cases died from suicide within 2-4 days of their last contact. For details, see Table 1.

For peer review only - http://bmjopen.bmj.com/site/about/guidelines.xhtml

For peer review only

Table 1. Characteristics of the suicide cases reported to the supervisory authority across the three cohorts. The data in the table comprise numbers and percentages, n (%).

		Cohort 1 (n = 279)	Cohort 2 (n = 436)	Cohort 3 (n = 316)
**Characteristic**				
**Age, years**	Range	15-95	13-93	11-95
	Percentile 25	36	33	29
	Percentile 50	50	49	42
	Percentile 75	64	61	57
**Gender**	Men	166 (60)	283 (65)	213 (67)
	Women	113 (40)	152 (35)	103 (33)
**Reporting healthcare service**	Psychiatric care	195 (70)	290 (67)	233 (74)
	Primary care	47 (17)	94 (22)	56 (18)
	Somatic care	21 (7)	33 (8)	16 (5)
	Other	16 (6)	18 (4)	11 (3)
**Days between last contact with healthcare services and death**	Range	0-70	0-88	0-240
	Percentile 25	0	1	0
	Percentile 50	2	4	3
	Percentile 75	7	10	9
**Receiving inpatient care at time of death**		45 (16)	36 (8)	44 (14)
**Receiving compulsory psychiatric treatment at the time of death***		15 (5)	22 (5)	20 (6)
**Major psychiatric diagnosis documented and coded in accordance with ICD-10 in patients' records**	Total (F00-F98)	228 (82)	371 (85)	288 (91)
	Affective disorder (F30)	119 (43)	153 (35)	105 (33)
	Anxiety disorder (F40)	35 (13)	77 (18)	60 (19)
	Substance abuse (F10)	29 (10)	51 (12)	37 (12)
	Psychosis (F20)	22 (8)	36 (8)	30 (10)
	Personality disorder (F60)	12 (4)	13 (3)	13 (4)
	Attention deficit disorder (F90)	1 (0)	13 (3)	12 (4)
	Autism spectrum (F84)	3 (1)	13 (3)	9 (3)
	Other	7 (2)	15 (3)	22 (7)
**Suicide-risk assessment documented in patients' records in the three months before death**	Absent	135 (49)	108 (25)	119 (38)
	Low	61 (22)	171 (39)	91 (29)
	Elevated, not acute	61 (22)	116 (27)	75 (24)
	High/acute	19 (7)	41 (9)	31 (10)
**Prior suicide attempt**		120 (46)	204 (47)	154 (49)
**Suicide method**	Hanging	112 (40)	160 (37)	128 (41)
	Intoxication	42 (15)	110 (25)	53 (17)

For peer review only - http://bmjopen.bmj.com/site/about/guidelines.xhtml

	Jumping	21 (8)	13 (3)	19 (6)
	Train	11 (4)	35 (8)	22 (7)
	Drowning	15 (5)	28 (6)	13 (4)
	Shooting	10 (4)	27 (6)	14 (4)
	Others	13 (8)	12 (3)	16 (5)
	Not reported	51 (18)	50 (12)	51 (16)
**Location of suicide**	Home	154 (56)	248 (57)	161 (51)
	Hospital	23 (8)	22 (5)	33 (10)
	Other	53 (19)	131 (30)	83 (26)
	Not reported	44 (16)	35 (8)	39 (12)

Note: Cohort 1: cases reported in 2006-2007, cohort 2: cases reported in 2015, and cohort 3: cases reported in 2017-2019. ICD-10: International Classification of Diseases and related Health Problems, 10th revision.

* includes both in-patient and out-patient compulsory treatment

For peer review only

Deficiencies in healthcare

Cohort 3 showed the largest proportion of cases for which deficiencies in healthcare were considered to have contributed to the suicide. In this cohort, only suicide cases considered to involve severe patient harm could have been prevented if different actions had been taken by healthcare professionals were to be reported. Over time, some changes in the proportions for the categories of deficiencies were observed, but they remained centered on final patient contact with healthcare services. In cohort 1 and 2, the most common deficiencies concerned "suicide risk assessment." In general, in cohort 1 these deficiencies related to an absence of local guidelines for suicide risk assessment, and in cohort 2 to non-adherence to existing guidelines. In cohort 3, deficiencies in "treatment" and "external communication" were the most common. Examples of deficiencies in "treatment" were delayed, or a lack of, follow-up after prescription of medication, or non-adherence to treatment guidelines. Examples of deficiencies in "external information" were a lack of or insufficient information exchange between healthcare providers. For details, see Table 2.

Table 2. Proportions of cases with deficiencies, as reported in the post-suicide investigations of the healthcare services' actions. The data in the table comprise numbers and percentages, n (%).

	**Cohort 1 (n = 279)**	**Cohort 2 (n = 436)**	**Cohort 3 (n = 316)**
**Cases with deficiencies, total**	136 (49)	240 (55)	248 (78)
**Category**			
**Communication and information**			
Communication with peers and family members	8 (3)	51 (12)	39 (12)
Documentation	57 (20)	65 (15)	68 (22)
External communication	21 (8)	74 (17)	91 (29)
Internal communication	18 (7)	61 (14)	68 (22)
**Education and competence**			
Education and competence not specified	12 (4)	54 (11)	50 (16)
Education and competence in suicide risk assessment	5 (2)	9 (2)	13 (4)

For peer review only - http://bmjopen.bmj.com/site/about/guidelines.xhtml

**Organization and management**			
Human resources	15 (5)	60 (14)	53 (17)
Number of beds	2 (1)	9 (2)	5 (2)
Organization/management	2 (1)	13 (3)	13 (4)
**Policies and procedures**			
Treatment	26 (9)	84 (19)	92 (29)
Suicide risk assessment	92 (33)	86 (20)	76 (24)
Work process	20 (7)	50 (11)	51 (16)
Diagnostics	16 (6)	54 (12)	41 (13)
Care plan and crisis plan	10 (4)	46 (11)	53 (17)
**Technics and equipment**	5 (2)	13 (3)	15 (5)
**Other**	2 (1)	11 (3)	0 (0)

Note. Cohort 1: cases reported in 2006-2007, cohort 2: cases reported in 2015, and cohort 3: cases reported in 2017-2019.

Proposed actions for addressing deficiencies

In a majority of the cases, the providers proposed actions for improving the healthcare services. The proportions of the action categories differed between the cohorts. In cohort 1, actions relating to "suicide risk assessment" were most common, usually involving the creation of new local guidelines regarding this issue. In cohorts 2 and 3, actions centered on education, present in more than half of the cases. Examples of educational actions were reminding staff about existing local guidelines, holding case-report discussions at staff meetings, and staging lectures regarding suicide risk assessment. For details, see Table 3.

Table 3. Proportions of cases for which actions were recommended in the post-suicide investigations. The data in the table comprise numbers and percentages, n (%).

	**Cohort 1 (n = 279)**	**Cohort 2 (n =436)**	**Cohort 3 (n = 316)**
**Cases with actions, total**	133 (48)	346 (79)	283 (90)
**Category**			
**Communication and information**			
Communication with peers and family	12 (4)	51 (12)	27 (9)
Documentation	39 (14)	71 (16)	65 (21)
External communication	22 (8)	80 (18)	83 (26)
Internal communication	15 (5)	55 (13)	46 (15)
**Education and competence**			
Education and competence not specified	35 (13)	166 (38)	136 (43)
Education and competence in suicide risk assessment	44 (16)	136 (31)	85 (27)
**Organization and management**			
Human resources	7 (3)	67 (15)	42 (13)
Number of beds	1 (0)	4 (1)	1 (0)
Organization/management	6 (2)	22 (5)	20 (6)
**Policies and procedures**			

For peer review only - http://bmjopen.bmj.com/site/about/guidelines.xhtml

For peer review only

Treatment	21 (8)	56 (13)	64 (20)
Suicide risk assessment	74 (27)	94 (22)	51 (16)
Work process	28 (10)	119 (27)	87 (28)
Diagnostics	8 (3)	28 (6)	25 (8)
Care plan and crisis plan	6 (2)	46 (11)	51 (16)
**Technics and equipment**	12 (4)	22 (5)	22 (7)
**Other**	1 (0)	8 (2)	3 (1)

Note. Cohort 1 comprises of cases reported in 2006-2007, cohort 2 cases reported in 2015, and cohort 3 cases reported in 2017-2019.

Learning and sharing

Any lessons learned and the sharing of experiences obtained from cases and investigations usually remained within the department in question. Sharing outside the department was reported in 4% (n = 17) of the cases in cohort 2, and in 7% (n = 21) of the cases in cohort 3. Sharing outside the department was not reported in any cases in cohort 1.

Routines

Over time, proposals for actions concerning updating or developing new routines became more common in the investigations. In cohorts 2 and 3, there were significantly more cases featuring the proposed development of new routines when compared with the number of cases for which an absence of routines was identified. In all cohorts, the number of revisions exceeded the number of identified dysfunctional routines. Non-adherence to existing routines was highlighted in almost one-third of the cases in cohort 3. For details, see Table 4.

Table 4. Deficiencies and actions in routines, reported in the post-suicide investigations.

		**Cohort 1 (n=279)**	**Cohort 2 (n=436)**	**Cohort 3 (n=316)**
**Routines, deficiencies**	Non-adherence	10 (4)	44 (10)	95 (30)
	Absent	38 (14)	30 (7)	28 (9)
	Dysfunctional	1 (0)	0 (0)	8 (3)
**Routines, actions**	Revision	24 (9)	58 (13)	47 (15)
	New	55 (20)	94 (22)*	99 (31)*

Note. The data in the table comprise numbers and percentage, n (%). Cohort 1: cases reported in 2006-2007, cohort 2: cases reported in 2015, and cohort 3: cases reported in 2017-2019.

* Significantly more cases involved the development of new routines when compared with the number of absent routines, $p < 0.001$

Organizational hierarchy

For both deficiencies and proposed actions, the microsystem perspective remained dominant over the 13-year period. However, cohorts 2 and 3 showed a significant increase in the proportion of deficiencies and actions at the mesosystem level compared with cohort 1. No deficiencies were found at the macrosystem level. For details, see Table 5.

Examples of deficiencies at the microsystem level were inadequacies in doctors' prescriptions or in suicide-risk assessments. Examples of actions at the microsystem level were case discussions at staff meetings, lectures, and the development of new checklists. Deficiencies at the mesosystem level

For peer review only - http://bmjopen.bmj.com/site/about/guidelines.xhtml

included shortcomings in cooperation between the psychiatric clinic and somatic clinic, or inadequate communication between the hospital and primary care center. Examples of actions at the mesosystem level were alterations of procedures for communication or cooperation between different healthcare providers.

Table 5. Respective distributions of the highest organizational hierarchy levels for the deficiencies and actions associated with the cases. Only the highest level for each case is noted. The data in the table comprise numbers and percentages, n (%).

		Cohort 1	Cohort 2	Cohort 3
Organizational level, deficiencies	**Micro**	121 (90)	157 (65)	179 (73)
	**Meso**	13 (10)	83 (35)*	67 (27)*
	**Macro**	0 (0)	0 (0)	0 (0)
Organizational level, actions	**Micro**	115 (85)	225 (65)	206 (75)
	**Meso**	20 (15)	120 (35)*	70 (25)*
	**Macro**	0 (0)	1 (0)	0 (0)

Note. Cohort 1: cases reported in 2006-2007, cohort 2: cases reported in 2015, and cohort 3: cases reported in 2017-2019.

* Significantly larger proportion of cases with deficiencies or actions at the mesosystem level when compared to cohort 1, $p < 0.005$

Decisions of the supervisory authority

In all cohorts, the majority of the reports from the healthcare providers were approved by the supervisory authority without further requirements. Immediate approval was provided for 59% (n = 164) of the reports for cohort 1, 65% (n = 284) for cohort 2, and 59% (n = 186) for cohort 3. Meanwhile, inspections of the healthcare provider occurred for 9% (n = 25) of the cases in cohort 1, 6% (n = 25) of those in cohort 2 and 4% (n = 13) of those in cohort 3. A physician employed at the supervisory authority was involved in the decision-making for 89% (n = 249) of the cases in cohort 1, in 4% (n = 17) of the cases in cohort 2, and 13% (n = 40) of the cases in cohort 3.

DISCUSSION

This study explored changes in the outcomes of post-suicide investigations by healthcare services in cases reported as potential incidents of patient harm, adopting a 13-year perspective. Possible improvements for patient safety that could contribute to suicide-prevention were also examined in the context of these reports.

Over time the investigations generally and consistently focused on final patient contact, analyzing the immediate interface between the patient and staff from a microsystem level perspective.

The most common measures recommended for all cohorts were updating existing or developing new routines, and educational actions - potentially unsustainable, person-based. Sharing conclusions across departments was planned in only a small percentage of the cases. This similarity of investigation outcomes over the years, regardless of changes in legislation, suggests that the investigations were adapted to suit the structure of the authority report rather than specific incidents, and imply that no new service improvements or lessons are being identified.

The suicide rate in Sweden has not shown any obvious decline since the reporting of all suicide cases became mandatory,[28] and the healthcare-service deficiencies highlighted in these reports as being of significance continue to occur. In other words, despite several thousand investigations into healthcare

For peer review only - http://bmjopen.bmj.com/site/about/guidelines.xhtml

performance prior to suicides over the last few decades, aimed at identifying actions to improve healthcare for patients with suicidal tendencies, the same contributing factors remain.[29] This suggests that the actions taken to date have not been sufficient. A possible means of addressing this would be the systematic aggregation and analysis of trends through multiple investigations, which would help to conclusively identify recurrent deficiencies, and encouraging investigators to act as facilitators of organizational development instead of mandating single investigations.[30]

Most of the reported cases in this study had their last contact with a healthcare professional within days of their deaths. Two-thirds of the cases lacked a documented report of an elevated risk of suicide in the months before the death, and this persisted across cohorts, despite the strong focus in many of the analyzed investigations on actions related to suicide-risk assessment and education. Over the years, there has been a shift from reports of an absence of local policies for suicide-risk assessment to reports of non-adherence to existing policies for suicide-risk assessment. In the studied cohorts, only 7-10% of the patients were documented as being at high risk of suicide during the last months before death. The proportion of patients receiving compulsory psychiatric treatment at the time of suicide remained constant over the years, at 5-6%. This low proportion may indicate that, in most cases, compulsory psychiatric care fulfills its purpose and serves as a protective factor for patients.

Approximately half of the suicide victims in all cohorts had a documented prior suicide attempt; learning from cases of the successful treatment of patients who have survived prior suicidal crises could thus be of importance for improving suicide prevention in healthcare. However, such learning actions are not recommended in the Swedish reporting system, which is currently based on a Safety-I model; thus possible learning opportunities are not supported unless a Safety-II perspective is supplemented.[4]

Cohort 3 showed a higher proportion of deficiencies in "education and competence" when compared to cohorts 1 and 2. These deficiencies were often connected to deficiencies in "human resources" and "internal communication," suggesting difficulties in recruiting personnel with adequate competence, shortcomings in the introduction of new staff, and complications integrating locum doctors.

Deficiencies in "external communication" and "treatment" were present in almost one-third of the cases in cohort 3. This cohort showed a younger population with some higher degree of psychiatric diagnoses, which suggests that this was a more complex group with a need for support from different care providers, requiring external collaboration and, possibly, more complex treatment interventions.

In all cohorts, there was a pronounced focus on routines. Updating existing or developing new routines was the most common recommendation proposed in the investigations over the years. All cohorts, but most obviously cohort 3, showed a mismatch between the number of cases where an absence of routines was noted and the number of cases for which the development of new routines was recommended. Further, the number of revisions exceeded the number of identified dysfunctional routines. Non-adherence to existing routines was highlighted in almost one-third of the cases in cohort 3, and the solutions seemed to focus on creating new routines instead of ensuring adherence, preconditions, and usability. Notably, reflections on why adherence to existing routines failed from a system perspective were missing in the investigations. Changes or reimplementation of routines are person-based and have weak efficacy from a systemic perspective, but require less effort than strong actions on a systemic level.[31 32] The same concerns were present regarding educational actions, which were highlighted in over half of the cases in cohorts 2 and 3. The dominance of person-based actions at the microsystem level is not unique for the Swedish setting. Kellogg et al obtained the same findings in a review conducted in the US,[12] and other studies have reported that investigators complete their analyses after identifying human error, rather than proceeding to identify system-based problems.[33 34] Attributing issues to human error easily leads to person-based solutions, and creates a focus on what is possible rather than what is needed.[30]

For peer review only - http://bmjopen.bmj.com/site/about/guidelines.xhtml

Suicide locations and methods were similar in all cohorts, but were reported in less than 90% of the investigations in cohort 3. This was surprising, as these cases were regarded as representing incidents of severe patient harm, and analysis of the specific circumstances concerning the suicide should be of importance in regard to evaluating preventable factors.

The distribution of the supervisory authority's decisions remained similar over the years; most reports were approved without further arrangements. In a small number of cases, the authority made a site visit, but the frequency of such visits declined as time passed. Supervision can be a strong tool and incitement for improvement and development of healthcare services,[14] but the results in this study suggest that the authority did not avail of this. Mandatory reporting thus was determined to be a process of information transfer between healthcare providers and the authority, rather than a means of creating a participative improvement that enhances safety for patients with suicidal tendencies.

The overall aim of the incident-reporting system is to make healthcare safer, which presupposes learning. However, learning that extends beyond the staff involved in the incident requires information-sharing. The review of the reports in this study showed that sharing information between departments was planned in a low percentage of cases. Learning is a complex social and participative process that involves people actively reflecting on and organizing shared knowledge and practices.[8] Safety begins, rather than ends, with incident reports, and requires broad, in-depth, and high-quality investigations and careful planning and follow-up of the implementation of corrective actions to ensure they are sustainable over time.[35] To generate persistent knowledge and learning from cases, feedback should include more than a passive, brief report in a staff meeting that reminds of or notifies of the updating of a routine.

Suicide is usually the final outcome of several interacting factors over time, and only a small proportion of suicides are committed in hospitals.[36 37] Most suicides occur in the patient's home without any witnesses or staff; this makes suicide, as a case of patient harm, somewhat different from most other kinds of such incidents in healthcare. The requirements of the report to the authority are the same for all kinds of incidents, meaning the investigating process may be adapted to suit the standard template rather than the specific character of the incident. Analyzing the last contact with a healthcare professional from a microsystem level perspective is not sufficient to learn how healthcare can better help patients with suicidal tendencies. The investigation should integrate analysis of the suicidal process over time, including suicide-prevention tools. To advance this issue, a shift in investigations requirements and reports is needed, as well as more sophisticated infrastructures for investigation, learning, and sharing in healthcare services.

Limitations and strengths

All data were based on the healthcare providers' investigations and reports to the supervisory authority. The content in these reports is regulated by law; however, the quality of analysis differs and there still may have been additional shortcomings and inadequacies that were not mentioned in the reports or observed by the authority. Furthermore, there is no national taxonomy for the categorization of deficiencies and actions; a coding scheme created by the authors and used in a prior study was used. The category of "other" was used only in a few cases, suggesting that the categories in the coding scheme covered most of the reported deficiencies and actions.

The strengths of this study are that all investigations concerned the same kind of incident; suicides, and the data were population-based. Further, all data collection and categorization were conducted by only one researcher, who is a psychiatrist with experience working with patient safety issues; this ensured a high level of consistency.

Conclusions

For peer review only - http://bmjopen.bmj.com/site/about/guidelines.xhtml

The mandatory reporting of suicides as potential cases of patient harm was shown to be restricted to information transfer between healthcare providers and the supervisory authority, rather than fostering participative improvement of patient safety for suicidal patients.

The similarity in outcomes across the cohorts, regardless of changes in legislation, suggests that the investigations were adapted to suit the structure of the authority's reports rather than the specific incident type, and that no new service improvements or lessons are being identified.

To develop more sophisticated infrastructures for investigation, learning, and information-sharing, it is necessary to learn more about preconditions and complexity in the analysis of suicides and the suicidal process.

A shift in investigations' recommendations and reports should be encouraged, to also include learning from successfully treated and resolved suicide-related crises.

Acknowledgements

The authors are grateful to Region Jönköpings county and Futurum for funding and to Public Health Agency of Sweden for support.

Authors´ contributor statement

EF designed the study, collected and registered the data, made the first analyses and wrote the manuscript. BAG, AR and ÅW contributed to the study design, analyses of the data and revisions of the manuscript. All authors read and approved the final manuscript.

Competing interests

The authors declare that they have no competing interests.

Funding

This study was funded by Futurum, the research center at Region Jönköping county.

Data sharing statement

The complete coding scheme is available by e-mailing elin.froding@rjl.se.

For peer review only

REFERENCES

1 Kohn LT CJ, Donaldson MS. To err is human: building a safer health system. Wahington DC: National Academies Press 2000.

2 Leape LL. Reporting of adverse events. *N Engl J Med* 2002;347(20):1633-8. doi: 10.1056/NEJMNEJMhpr011493.

3 Braithwaite J, Wears RL, Hollnagel E. Resilient health care: turning patient safety on its head. *Int J Qual Health Care* 2015;27(5):418-20. doi: 10.1093/intqhc/mzv063.

4 Hollnagel E, Wears RL, Braithwaite J. From Safety-I to Safety-II: a white paper. *Published simultaneously by the University of Southern Denmark, University of Florida, USA, and Macquarie University, Australia: The Resilient Helath Care Net* 2015.

For peer review only - http://bmjopen.bmj.com/site/about/guidelines.xhtml

5 Stanhope N, Crowley-Murphy M, Vincent C*, et al.* An evaluation of adverse incident reporting. *J Eval Clin Pract* 1999;5(1):5-12. doi: 10.1046/j.1365-2753.1999.00146.x.

6 Vincent C, Amalberti R. Safety in healthcare is a moving target. *BMJ Qual Saf* 2015;24(9):539-40. doi: 10.1136/bmjqs-2015-004403.

7 Macrae C, Vincent C. Learning from failure: the need for independent safety investigation in healthcare. *J R Soc Med* 2014;107(11):439-43. doi: 10.1177/0141076814555939

8 Macrae C. The problem with incident reporting. *BMJ Qual Saf* 2016;25(2):71-5. doi: 10.1136/bmjqs-2015-004732.

9 Macrae C. Remembering to learn: the overlooked role of remembrance in safety improvement. *BMJ Qual Saf* 2017;26(8):678-82. doi: 10.1136/bmjqs-2016-005547

10 Wrigstad J, Bergström J, Gustafsson P. Mind the gap between recommendation and implementation—principles and lessons in the aftermath of incident investigations: a semi-quantitative and qualitative study of factors leading to the successful implementation of recommendations. *BMJ Open* 2014;4 doi: 10.1136/bmjopen-2014-005326.

11 Roos af Hjelmsäter E, Ros A, Gäre BA*, et al.* Deficiencies in healthcare prior to suicide and actions to deal with them: a retrospective study of investigations after suicide in Swedish healthcare. *BMJ Open* 2019;9(12):e032290. doi: 10.1136/bmjopen-2019-032290.

12 Kellogg KM, Hettinger Z, Shah M*, et al.* Our current approach to root cause analysis: is it contributing to our failure to improve patient safety? *BMJ Qual Saf* 2017;26(5):381-87. doi: 10.1136/bmjqs-2016-005991.

13 Mitchell I, Schuster A, Smith K*, et al.* Patient safety incident reporting: a qualitative study of thoughts and perceptions of experts 15 years after 'To Err is Human'. *BMJ Qual Saf* 2016;25(2):92-9. doi: 10.1136/bmjqs-2015-004405.

14 Leistikow I, Mulder S, Vesseur J*, et al.* Learning from incidents in healthcare: the journey, not the arrival, matters. *BMJ Qual Saf* 2017;26(3):252-56. doi: 10.1136/bmjqs-2015-004853.

15 Shojania KG, Thomas EJ. Trends in adverse events over time: why are we not improving? *BMJ Qual Saf* 2013;22(4):273-77. doi: 10.1136/bmjqs-2013-001935.

16 The Swedish Patient Safety Act (SFS 2010:659) [https://www.riksdagen.se/sv/dokument-lagar/dokument/svensk-forfattningssamling/patientsakerhetslag-2010659_sfs-2010-659]. [accessed 2 February 2019].

17 World Health Organization. Suicide data 2018. Available from: http://www.who.int/mental_health/prevention/suicide/suicideprevent/en/ [accessed 17 October 2018].

18 Luoma JB, Martin CE, Pearson JL. Contact with mental health and primary care providers before suicide: a review of the evidence. *Am J Psychiatry* 2002;159(6):909-16. doi: 10.1176/appi.ajp.159.6.909.

19 Chock MM, Lin JC, Athyal VP*, et al.* Differences in health care utilization in the year before suicide death: a population-based case-control ctudy. *Mayo Clin Proc* 2019;94(10):1983-93. doi: 10.1016/j.mayocp.2019.04.037.

20 Cavanagh JT, Carson AJ, Sharpe M*, et al.* Psychological autopsy studies of suicide: a systematic review. *Psychol Med* 2003;33(3):395-405.

21 Zalsman G, Hawton K, Wasserman D*, et al.* Suicide prevention strategies revisited: 10-year systematic review. *Lancet Psychiatry* 2016;3(7):646-59. doi: 10.1016/s2215-0366(16)30030-x.

22 Wasserman D, Rihmer Z, Rujescu D*, et al.* The European psychiatric association (EPA) guidance on suicide treatment and prevention. *Eur Psychiatry* 2012;27(2):129-41. doi: 10.1016/j.eurpsy.2011.06.003.

23 Mann JJ, Apter A, Bertolote J*, et al.* Suicide prevention strategies: a systematic review. *JAMA* 2005;294(16):2064-74. doi: 10.1001/jama.294.16.2064.

24 The National Board of Health and Welfare. Socialstyrelsens föreskrifter om anmälan av vårdskador enligt lex Maria (SOSFS 2005:28)[The National Board of Health and Welfare's regulations on the reporting of severe patient harm]. Stockholm: The National Board of Health and Welfare.

For peer review only - http://bmjopen.bmj.com/site/about/guidelines.xhtml

25 The National Board of Health and Welfare. Socialstyrelsens föreskrifter och allmänna råd om vårdgivares systematiska patientsäkerhetsarbete (HSLF-FS 2017:40) [The National Board of Health and Welfare's regulations and general advice on healthcare organization´s systematic patient safety work]. Stockholm: The National Board of Health and Welfare.
26 Swedish Association of Local Authorities and Regions. Risk och händelseanalys [Risk analysis and adverse events analysis]. Stockholm: Swedish Association of Local Authorities and Regions 2015.
27 Nelson EC, Batalden PB, Godfrey MM. Quality by design: a clinical microsystems approach. San Fransisco: Jossey-Bass 2007.
28 The National Board of Health and Welfare. Statistikdatabas för dödsorsaker [Statistical databease, cause of death]: The National Board of Health and Welfare; 2020. Available from: https://sdb.socialstyrelsen.se/if_dor/resultat.aspx [accessed 2020-02-02].
29 Health and Social Care Inspectorate. https://www.ivo.se/publicerat-material/statistik/lex-maria-och-lex-sarah/ [accessed 2020-02-05].
30 Nicolini D, Waring J, Mengis J. Policy and practice in the use of root cause analysis to investigate clinical adverse events: mind the gap. *Soc Sci Med* 2011;73(2):217-25. doi: 10.1016/j.socscimed.2011.05.010.
31 Trbovich P, Shojania KG. Root-cause analysis: swatting at mosquitoes versus draining the swamp. *BMJ Qual Saf* 2017;26(5):350-53. doi: 10.1136/bmjqs-2016-006229.
32 Cafazzo JA, St-Cyr O. From discovery to design: the evolution of human factors in healthcare. *Healthc Q* 2012;15(April (Special Issue)):24-29.
33 Mills PD, Neily J, Luan D*, et al.* Actions and implementation strategies to reduce suicidal events in the Veterans Health Administration. *Jt Comm J Qual Patient Saf* 2006;32(3):130-41.
34 Percarpio KB, Watts BV, Weeks WB. The effectiveness of root cause analysis: what does the literature tell us? *Jt Comm J Qual Patient Saf* 2008;34(7):391-8. doi: 10.1016/s1553-7250(08)34049-5.
35 Macrae C. Close calls: managing risk and resilience in airline flight safety: Springer 2014.
36 Qin P, Nordentoft M. Suicide risk in relation to psychiatric hospitalization: evidence based on longitudinal registers. *Arch Gen Psychiatry* 2005;62(4):427-32. doi: 10.1001/archpsyc.62.4.427.
37 Ballard ED PM, Henderson D, et al. Suicide in the medical setting. *Jt Comm J Qual Patient Saf* 2008;34:474-81.

For peer review only

For peer review only - http://bmjopen.bmj.com/site/about/guidelines.xhtml

BMJ Open

Suicide as an incident of severe patient harm – a retrospective review of investigations of the healthcare provided to patients prior to their suicide

Journal:	*BMJ Open*
Manuscript ID	bmjopen-2020-044068.R1
Article Type:	Original research
Date Submitted by the Author:	02-Feb-2021
Complete List of Authors:	Fröding, Elin; Jönköping University; Region Jönköpings län<br>Gäre, Boel Andersson; Jönköping University; Region Jönköpings län, Futurum<br>Westrin, Åsa; Lund University Department of Clinical Sciences Malmo, Division of Psychiatry, Lund University ; Psychiatry Research Skåne, Office for Psychiatry and Habilitation, Region Skåne<br>Ros, Axel; Jönköping University; Region Jönköpings län
**Primary Subject Heading**:	Health services research
Secondary Subject Heading:	Mental health
Keywords:	Health & safety < HEALTH SERVICES ADMINISTRATION & MANAGEMENT, Quality in health care < HEALTH SERVICES ADMINISTRATION & MANAGEMENT, Risk management < HEALTH SERVICES ADMINISTRATION & MANAGEMENT, Suicide & self-harm < PSYCHIATRY

SCHOLARONE™
Manuscripts

BMJ

I, the Submitting Author has the right to grant and does grant on behalf of all authors of the Work (as defined in the below author licence), an exclusive licence and/or a non-exclusive licence for contributions from authors who are: i) UK Crown employees; ii) where BMJ has agreed a CC-BY licence shall apply, and/or iii) in accordance with the terms applicable for US Federal Government officers or employees acting as part of their official duties; on a worldwide, perpetual, irrevocable, royalty-free basis to BMJ Publishing Group Ltd ("BMJ") its licensees and where the relevant Journal is co-owned by BMJ to the co-owners of the Journal, to publish the Work in this journal and any other BMJ products and to exploit all rights, as set out in our licence.

The Submitting Author accepts and understands that any supply made under these terms is made by BMJ to the Submitting Author unless you are acting as an employee on behalf of your employer or a postgraduate student of an affiliated institution which is paying any applicable article publishing charge ("APC") for Open Access articles. Where the Submitting Author wishes to make the Work available on an Open Access basis (and intends to pay the relevant APC), the terms of reuse of such Open Access shall be governed by a Creative Commons licence – details of these licences and which Creative Commons licence will apply to this Work are set out in our licence referred to above.

Other than as permitted in any relevant BMJ Author's Self Archiving Policies, I confirm this Work has not been accepted for publication elsewhere, is not being considered for publication elsewhere and does not duplicate material already published. I confirm all authors consent to publication of this Work and authorise the granting of this licence.

For peer review only

Title page

Suicide as an incident of severe patient harm – a retrospective review of investigations of the healthcare provided to patients prior to their suicide

Authors

PhD. Elin Fröding, Prof. Boel Andersson Gäre, Prof. Åsa Westrin, MD. Axel Ros

Corresponding author

Name and address: Elin Fröding, Höglandssjukhuset Hus 34 pl 4, 575 81 Eksjö, Sweden

Mail and phone number: Elin.froding@rjl.se, +4670-3577396

Co-authors

Prof. Boel Andersson Gäre, Jönköping University, Jönköping, Sweden and Futurum, Region Jönköping County, Sweden.

Prof. Åsa Westrin, Department of Clinical Sciences Lund, Psychiatry, Lund University, Sweden and Office for Psychiatry and Habilitation, Psychiatry Research Skåne, Region Skåne, Sweden.

MD. Axel Ros, Jönköping University, Jönköping, Sweden

Key words

Health & safety, patient safety, risk management, quality in health care, suicide

Word count

4193

For peer review only - http://bmjopen.bmj.com/site/about/guidelines.xhtml

ABSTRACT

Objectives

The reporting of suicides among recipients of healthcare services to the supervisory authority was mandatory in Sweden between 2006-2017. This study, adopting a 13-year perspective, aimed to explore how this mandatory reporting has influenced associated investigations conducted by the healthcare services, and also examined the lessons obtained, and whether any suicide-prevention-related improvements in terms of patient safety had followed.

Design and settings

This is a retrospective study of reports from Swedish primary and secondary healthcare after suicide to the regulatory authority in Sweden. Three cohorts of suicide cases, each from a different time period, were chosen for analysis. Complete reports of the incident investigations conducted by the healthcare providers with associated patient records and the subsequent decisions of the supervisory authority were analyzed by using a coding scheme.

Results

The investigations largely adopted a microsystem perspective, focusing on final patient contact, throughout the overall study period. Updating existing or developing new routines as well as educational actions had been increasingly proposed, while sharing conclusions across departments was rarely recommended.

Conclusions

The mandatory reporting of suicides as potential cases of patient harm was shown to be restricted to information transfer between healthcare providers and the supervisory authority, rather than fostering participative improvement of patient safety for suicidal patients.

The similarity in outcomes across the cohorts, regardless of changes in legislation, suggests that the investigations were adapted to suit the structure of the authority's reports rather than the specific incident type, and that no new service improvements or lessons are being identified.

To develop more sophisticated infrastructures for investigation, learning, and information-sharing, it is necessary to learn more about preconditions and complexity in the analysis of suicides and the suicidal process.

A shift in investigations' recommendations and reports should be encouraged, to also include learning from successfully treated and resolved suicide-related crises.

Strengths and limitations of the study

- To our knowledge, this is the first evaluation of the outcomes of investigations of specific types of patient harm over time, here exemplified by suicide.
- All investigations concerned the same kind of incident; suicides, and the data were population-based.

For peer review only - http://bmjopen.bmj.com/site/about/guidelines.xhtml

- All data were based on the healthcare providers' investigations and reports to the supervisory authority, the content in these reports is regulated by law; however, the quality of analysis differs, which was not evaluated in this study.
- All data collection and categorization were conducted by only one researcher, which rendered categorization vulnerable to bias; however this ensured a high level of consistency.

BACKGROUND

Deaths that occur as a result of patient harm represent a contrast to healthcare services' aim of a high level of patient safety, and such incidents can serve as powerful motivators for learning and improvement.[1 2] In recent decades, efforts to increase patient safety have been intensified. In particular, the reporting and investigating of cases of severe patient injury in order to identify risks and improve patient safety have become widespread safety-improvement strategies.[2] This reflects a safety-I perspective regarding patient safety, with focus on incidents that could have or did lead to harm for patients during healthcare, assuming that safety is achieved by eliminating what can go wrong.[3] This perspective assumes that adverse outcomes are caused by identifiable failures or malfunctions of specific components different from situations when things go right.[3 4] Similarly, root cause analysis (RCA) has become one of the most widespread tools used in the investigation of healthcare-related incidents, and presumes that such incidents can be explained by linear cause-effect chains.[5 6] Determining what had happened and why an incident occurred should not be the final goal of an incident investigation; the identification of gaps in service provision and means of improving relevant areas of the healthcare organization are important for improving safety.[7] To successfully learn from past incidents, methods to sustainably record and share relevant data are essential.[8 9] However, prior studies have shown that, in healthcare, post-incident investigations usually provide little learning beyond the staff and units involved.[10 11] Thus, the actual value of incident-reporting systems and the RCA approach in healthcare has been questioned.[8 12-15] With the introduction of new concepts in patient safety, such as safety-II and resilient healthcare, new approaches for improving healthcare have focused on learning from all occurrences in daily practice: to identify both those factors that support a good outcome and those that increase the risk of patient harm.[3 4] In the concept of safety-II, focus is on "work in practice", i. e. to better understand how clinicians provide good quality healthcare in real-time dynamic systems, including the interactions between patient care, environmental contexts, and healthcare culture. In this perspective, safety is achieved through understanding health-care staff's adaptations to varying conditions and ensuring that as much as possible goes well.

Swedish law states that events with severe patient harm, as well as events involving risk of severe patient harm, that could have been avoided if appropriate actions had been taken by healthcare professionals, should be reported to the supervisory authority.[16] This report to the authority should be preceded by an investigation, conducted by the healthcare providing organization, of the healthcare services provided to the patient before the adverse event. The content of the investigation is regulated by law, and requires identification of the contributory causes of the incident and of service improvements that may prevent the reoccurrence of such an incident.

Suicide is a global health problem with an estimated 800 000 deaths worldwide every year.[17] Suicidal behaviours are heterogeneous and complex and influenced by several interacting biological, genetic, psychological, social, environmental and situational factors over time.[18] A large proportion of the individuals who die from suicide have contact with healthcare professionals close in time before their deaths.[19 20] Post-suicide studies have found that the vast majority of suicide victims have psychiatric illnesses at the time of their deaths.[21 22 23] This suggests that healthcare professionals play an important role in suicide prevention.[24] However, the nature of suicide as a process going on over time, usually occurring outside the hospitals without any witnesses nor staff around, make suicide as a case of patient harm, somewhat different from most other kinds of such incidents in healthcare. Few studies

For peer review only - http://bmjopen.bmj.com/site/about/guidelines.xhtml

have applied patient safety paradigms to advance understanding of preventing suicide[25] although there are examples of studies of health services associated with reductions in suicide rates, such as well-developed community outpatient services[26] and the implementation of 24-hour crisis services.[27] Kapur et al. suggest system-wide changes implemented across the patient care pathway could be a key strategy for improving patient safety in mental health care.[28]

In an effort to understand whether failures in any area of the healthcare system have contributed to suicide, and in an attempt to improve suicide-prevention, the Swedish National Board of Health and Welfare in 2006 stipulated that all suicides that occur among patients who were receiving healthcare or were in contact with healthcare services within the four weeks preceding the event must be reported to the authority by the healthcare provider.[29] This remained mandatory regardless of whether the provider determined the suicide to be preventable. In September 2017, this regulation was updated to state that only suicides regarded as "severe patient harm" (i.e., preventable) must be reported to the supervisory authority.[30]

Before 2011, the supervisory authority performed their own investigations of incidents, and had the power to reprimand the provider and responsible staff. The role of the supervisory authority changed in 2011, when the Swedish Patient Safety Act (2010:659)[16] was implemented. This new law made healthcare organizations responsible for patient-safety improvement, and the role of the supervisory authority was changed to review the investigations made by the providers, and ensure that they were satisfactorily fulfilled and that appropriate actions had been taken to ensure a high level of patient safety. In particular, the authority determines whether the healthcare provider has fulfilled their legislated duties, or whether there are shortcomings in the investigation, in which case the authority may recommend revisions or conduct a site visit to inspect the healthcare provider.

To our knowledge, there are no published evaluations of the outcomes of investigations of specific types of patient harm over time, here exemplified by suicide.

The objective of this study was to explore how mandatory reporting of suicide cases as incidents of potential patient harm has influenced the investigations of healthcare systems. To perform this, a 13-year perspective was adopted, and the lessons and possible improvements for patient safety regarding suicide prevention were examined.

METHODS

Cases

Three cohorts of suicide cases, each from a different time period, that were reported to the supervisory authority were chosen for analysis. Cohort 1 comprised the cases reported to the supervisory authority in 2006, from the time the reporting of suicides became mandatory, to 2007 (n = 279). Cohort 2 comprised all suicides reported in 2015, this represented a period when mandatory reporting was well-established among healthcare providers (n = 436). Cohort 3 comprised all reported suicides from September 1, 2017, which was the time the law regarding reporting was changed, to November 30, 2019 (n = 316).

Complete reports of the incident investigations conducted by the healthcare providers with associated patient records and the subsequent decisions of the supervisory authority were obtained from the supervisory authority. Every individual suicide case was given a code number and the patient's demographic data and treatment received in the months preceding his/her death were registered. Major diagnoses were documented and coded in accordance with the International Statistical Classification of Diseases and related Health Problems, 10th revision (i.e., ICD-10).

Categorization of data

For peer review only - http://bmjopen.bmj.com/site/about/guidelines.xhtml

A coding scheme was used to categorize the contributory causes of the respective suicides, the actions reported in the investigations and the decisions of the authority. The same coding scheme was used in a prior study of reported suicide cases in Sweden.[11] This scheme is based on the general categories used in the most common method of investigating adverse events in Swedish healthcare, which is in turn based on RCA.[31] To make the categorization more specific, four of the major categories were divided into additional subcategories. Every category was described and exemplified and a category of "others" was added in case none of the other categories was considered appropriate. In this present study, the contributory causes were reported as "deficiencies." Meanwhile, an "action" was defined as any intervention performed in attempt to prevent new suicides: therefor, actions taken to prevent reported suicides (telephone calls, resuscitations) or actions aimed at informing family members or staff that a suicide had occurred were not registered as actions in this study. Separate notes were made when a deficiency or action was related to a healthcare-service routine, as well as in regard to how learning from the investigation was described. To ensure consistency, all data collection and categorization were conducted by only one researcher (EF), a psychiatrist with extensive experience in patient-safety issues.

Organizational levels

Classification of the organizational levels of deficiencies and actions was conducted to better understand where in the organizational system the identified deficiencies and actions were situated. The deficiencies and actions were coded based on a micro-meso-macro-perspective.[32] Microsystems were defined as the basic elements of the healthcare services provided for the patient, such as the inpatient or outpatient care unit. The mesosystem encompassed interactions between different microsystem units, such as cooperation between departments or different healthcare providers. The macrosystem involved the entire healthcare system, such as legislation, political prioritizations, and national policies on healthcare. For each case, the highest organizational level for each deficiency and action was coded.

Supervisory authority

The mandate stipulated to the authority by legislation differed between cohort 1 and cohorts 2 and 3, hence the formulation of the decisions also differed. In this paper, to facilitate comparison among these outcomes, for all cohorts only decisions categorized as "immediate approval" and "inspection" were noted, as these remained unchanged. A note was made if a physician employed by the supervisory authority was involved in the decision-making.

Statistical analyses

Frequencies for each category, organizational hierarchal level of deficiencies and actions, and decisions of the supervisory authority were analyzed per individual and aggregated per cohort.

Chi-square tests of independence were used to compare the number of new routines and the absence of routines within the same cohort, as well as the proportion of the organizational hierarchy of deficiencies and actions between cohorts. We considered a two-sided p value of < 0.05 to indicate statistical significance. As the pre-requisites differed between the cohorts, no further statistical analyses to compare the cohorts were judged to be possible.

The statistical analyses were performed using IBM SPSS Statistics 24.

Ethical review

According to the Swedish *Act Concerning the Ethical Review of Research Involving Humans* (2003:460) and an advisory opinion from the Regional Ethical Review Board (no. 2017/234), this study did not require an ethical review as it did not include human participants.

For peer review only - http://bmjopen.bmj.com/site/about/guidelines.xhtml

Patient and Public Involvement

Patients or public were not involved in this study.

RESULTS

Cases

Demographic data for the cases showed similarities across the cohorts, with a dominance of men and a majority of cases reported by psychiatric care. One-fourth of the cases died from suicide within one day of their last contact with a healthcare professional; half of the cases died from suicide within 2-4 days of their last contact. For details, see Table 1.

Table 1. Characteristics of the suicide cases reported to the supervisory authority across the three cohorts. The data in the table comprise numbers and percentages, n (%).

		Cohort 1 (n = 279)	Cohort 2 (n = 436)	Cohort 3 (n = 316)
**Characteristic**				
**Age, years**	Range	15-95	13-93	11-95
	Percentile 25	36	33	29
	Percentile 50	50	49	42
	Percentile 75	64	61	57
**Gender**	Men	166 (60)	283 (65)	213 (67)
	Women	113 (40)	152 (35)	103 (33)
**Reporting healthcare service**	Psychiatric care	195 (70)	290 (67)	233 (74)
	Primary care	47 (17)	94 (22)	56 (18)
	Somatic care	21 (7)	33 (8)	16 (5)
	Other	16 (6)	18 (4)	11 (3)
**Days between last contact with healthcare services and death**	Range	0-70	0-88	0-240
	Percentile 25	0	1	0
	Percentile 50	2	4	3
	Percentile 75	7	10	9
**Receiving inpatient care at time of death**		45 (16)	36 (8)	44 (14)
**Receiving compulsory psychiatric treatment at the time of death***		15 (5)	22 (5)	20 (6)
**Major psychiatric diagnosis documented and coded in accordance with ICD-10 in patients' records**	Total (F00-F98)	228 (82)	371 (85)	288 (91)
	Affective disorder (F30)	119 (43)	153 (35)	105 (33)
	Anxiety disorder (F40)	35 (13)	77 (18)	60 (19)
	Substance abuse (F10)	29 (10)	51 (12)	37 (12)
	Psychosis (F20)	22 (8)	36 (8)	30 (10)
	Personality disorder (F60)	12 (4)	13 (3)	13 (4)
	Attention deficit disorder (F90)	1 (0)	13 (3)	12 (4)

For peer review only - http://bmjopen.bmj.com/site/about/guidelines.xhtml

	Autism spectrum (F84)	3 (1)	13 (3)	9 (3)
	Other	7 (2)	15 (3)	22 (7)
**Suicide-risk assessment documented in patients' records in the three months before death**	Absent	135 (49)	108 (25)	119 (38)
	Low	61 (22)	171 (39)	91 (29)
	Elevated, not acute	61 (22)	116 (27)	75 (24)
	High/acute	19 (7)	41 (9)	31 (10)
**Prior suicide attempt**		120 (46)	204 (47)	154 (49)
**Suicide method**	Hanging	112 (40)	160 (37)	128 (41)
	Intoxication	42 (15)	110 (25)	53 (17)
	Jumping	21 (8)	13 (3)	19 (6)
	Train	11 (4)	35 (8)	22 (7)
	Drowning	15 (5)	28 (6)	13 (4)
	Shooting	10 (4)	27 (6)	14 (4)
	Others	13 (8)	12 (3)	16 (5)
	Not reported	51 (18)	50 (12)	51 (16)
**Location of suicide**	Home	154 (56)	248 (57)	161 (51)
	Hospital	23 (8)	22 (5)	33 (10)
	Other	53 (19)	131 (30)	83 (26)
	Not reported	44 (16)	35 (8)	39 (12)

Note: Cohort 1: cases reported in 2006-2007, cohort 2: cases reported in 2015, and cohort 3: cases reported in 2017-2019. ICD-10: International Classification of Diseases and related Health Problems, 10th revision.

* includes both in-patient and out-patient compulsory treatment

Deficiencies in healthcare

Cohort 3 showed the largest proportion of cases for which deficiencies in healthcare were considered to have contributed to the suicide. In this cohort, only suicide cases considered to involve severe patient harm could have been prevented if different actions had been taken by healthcare professionals were to be reported. Over time, some changes in the proportions for the categories of deficiencies were observed, but they remained centered on final patient contact with healthcare services. In cohort 1 and 2, the most common deficiencies concerned "suicide risk assessment." In general, in cohort 1 these deficiencies related to an absence of local guidelines for suicide risk assessment, and in cohort 2 to non-adherence to existing guidelines. In cohort 3, deficiencies in "treatment" and "external communication" were the most common. Examples of deficiencies in "treatment" were delayed, or a lack of, follow-up after prescription of medication, or non-adherence to treatment guidelines. Examples of deficiencies in "external information" were a lack of or insufficient information exchange between healthcare providers. For details, see Table 2.

Table 2. Proportions of cases with deficiencies, as reported in the post-suicide investigations of the healthcare services' actions. The data in the table comprise numbers and percentages, n (%).

	**Cohort 1 (n = 279)**	**Cohort 2 (n = 436)**	**Cohort 3 (n = 316)**

For peer review only - http://bmjopen.bmj.com/site/about/guidelines.xhtml

**Cases with deficiencies, total**	136 (49)	240 (55)	248 (78)
**Category**			
**Communication and information**			
Communication with peers and family members	8 (3)	51 (12)	39 (12)
Documentation	57 (20)	65 (15)	68 (22)
External communication	21 (8)	74 (17)	91 (29)
Internal communication	18 (7)	61 (14)	68 (22)
**Education and competence**			
Education and competence not specified	12 (4)	54 (11)	50 (16)
Education and competence in suicide risk assessment	5 (2)	9 (2)	13 (4)
**Organization and management**			
Human resources	15 (5)	60 (14)	53 (17)
Number of beds	2 (1)	9 (2)	5 (2)
Organization/management	2 (1)	13 (3)	13 (4)
**Policies and procedures**			
Treatment	26 (9)	84 (19)	92 (29)
Suicide risk assessment	92 (33)	86 (20)	76 (24)
Work process	20 (7)	50 (11)	51 (16)
Diagnostics	16 (6)	54 (12)	41 (13)
Care plan and crisis plan	10 (4)	46 (11)	53 (17)
**Technics and equipment**	5 (2)	13 (3)	15 (5)
**Other**	2 (1)	11 (3)	0 (0)

Note. Cohort 1: cases reported in 2006-2007, cohort 2: cases reported in 2015, and cohort 3: cases reported in 2017-2019.

Proposed actions for addressing deficiencies

In a majority of the cases, the providers proposed actions for improving the healthcare services. The proportions of the action categories differed between the cohorts. In cohort 1, actions relating to "suicide risk assessment" were most common, usually involving the creation of new local guidelines regarding this issue. In cohorts 2 and 3, actions centered on education, present in more than half of the cases. Examples of educational actions were reminding staff about existing local guidelines, holding case-report discussions at staff meetings, and staging lectures regarding suicide risk assessment. For details, see Table 3.

Table 3. Proportions of cases for which actions were recommended in the post-suicide investigations. The data in the table comprise numbers and percentages, n (%).

	**Cohort 1 (n = 279)**	**Cohort 2 (n =436)**	**Cohort 3 (n = 316)**
**Cases with actions, total**	133 (48)	346 (79)	283 (90)
**Category**			
**Communication and information**			
Communication with peers and family	12 (4)	51 (12)	27 (9)

For peer review only - http://bmjopen.bmj.com/site/about/guidelines.xhtml

Documentation	39 (14)	71 (16)	65 (21)
External communication	22 (8)	80 (18)	83 (26)
Internal communication	15 (5)	55 (13)	46 (15)
**Education and competence**			
Education and competence not specified	35 (13)	166 (38)	136 (43)
Education and competence in suicide risk assessment	44 (16)	136 (31)	85 (27)
**Organization and management**			
Human resources	7 (3)	67 (15)	42 (13)
Number of beds	1 (0)	4 (1)	1 (0)
Organization/management	6 (2)	22 (5)	20 (6)
**Policies and procedures**			
Treatment	21 (8)	56 (13)	64 (20)
Suicide risk assessment	74 (27)	94 (22)	51 (16)
Work process	28 (10)	119 (27)	87 (28)
Diagnostics	8 (3)	28 (6)	25 (8)
Care plan and crisis plan	6 (2)	46 (11)	51 (16)
**Technics and equipment**	12 (4)	22 (5)	22 (7)
**Other**	1 (0)	8 (2)	3 (1)

Note. Cohort 1 comprises of cases reported in 2006-2007, cohort 2 cases reported in 2015, and cohort 3 cases reported in 2017-2019.

Learning and sharing

Any lessons learned and the sharing of experiences obtained from cases and investigations usually remained within the department in question. Sharing outside the department was reported in 4% (n = 17) of the cases in cohort 2, and in 7% (n = 21) of the cases in cohort 3. Sharing outside the department was not reported in any cases in cohort 1.

Routines

Over time, proposals for actions concerning updating or developing new routines became more common in the investigations. In cohorts 2 and 3, there were significantly more cases featuring the proposed development of new routines when compared with the number of cases for which an absence of routines was identified. In all cohorts, the number of revisions exceeded the number of identified dysfunctional routines. Non-adherence to existing routines was highlighted in almost one-third of the cases in cohort 3. For details, see Table 4.

Table 4. Deficiencies and actions in routines, reported in the post-suicide investigations.

		**Cohort 1 (n=279)**	**Cohort 2 (n=436)**	**Cohort 3 (n=316)**
**Routines, deficiencies**	Non-adherence	10 (4)	44 (10)	95 (30)
	Absent	38 (14)	30 (7)	28 (9)
	Dysfunctional	1 (0)	0 (0)	8 (3)
**Routines, actions**	Revision	24 (9)	58 (13)	47 (15)
	New	55 (20)	94 (22)*	99 (31)*

For peer review only - http://bmjopen.bmj.com/site/about/guidelines.xhtml

Note. The data in the table comprise numbers and percentage, n (%). Cohort 1: cases reported in 2006-2007, cohort 2: cases reported in 2015, and cohort 3: cases reported in 2017-2019.

* Significantly more cases involved the development of new routines when compared with the number of absent routines, $p < 0.001$

Organizational hierarchy

For both deficiencies and proposed actions, the microsystem perspective remained dominant over the 13-year period. However, cohorts 2 and 3 showed a significant increase in the proportion of deficiencies and actions at the mesosystem level compared with cohort 1. No deficiencies were found at the macrosystem level. For details, see Table 5.

Examples of deficiencies at the microsystem level were inadequacies in doctors' prescriptions or in suicide-risk assessments. Examples of actions at the microsystem level were case discussions at staff meetings, lectures, and the development of new checklists. Deficiencies at the mesosystem level included shortcomings in cooperation between the psychiatric clinic and somatic clinic, or inadequate communication between the hospital and primary care center. Examples of actions at the mesosystem level were alterations of procedures for communication or cooperation between different healthcare providers.

Table 5. Respective distributions of the highest organizational hierarchy levels for the deficiencies and actions associated with the cases. Only the highest level for each case is noted. The data in the table comprise numbers and percentages, n (%).

		Cohort 1	Cohort 2	Cohort 3
Organizational level, deficiencies	**Micro**	121 (90)	157 (65)	179 (73)
	**Meso**	13 (10)	83 (35)*	67 (27)*
	**Macro**	0 (0)	0 (0)	0 (0)
Organizational level, actions	**Micro**	115 (85)	225 (65)	206 (75)
	**Meso**	20 (15)	120 (35)*	70 (25)*
	**Macro**	0 (0)	1 (0)	0 (0)

Note. Cohort 1: cases reported in 2006-2007, cohort 2: cases reported in 2015, and cohort 3: cases reported in 2017-2019.

* Significantly larger proportion of cases with deficiencies or actions at the mesosystem level when compared to cohort 1, $p < 0.005$

Decisions of the supervisory authority

In all cohorts, the majority of the reports from the healthcare providers were approved by the supervisory authority without further requirements. Immediate approval was provided for 59% (n = 164) of the reports for cohort 1, 65% (n = 284) for cohort 2, and 59% (n = 186) for cohort 3. Meanwhile, inspections of the healthcare provider occurred for 9% (n = 25) of the cases in cohort 1, 6% (n = 25) of those in cohort 2 and 4% (n = 13) of those in cohort 3. A physician employed at the supervisory authority was involved in the decision-making for 89% (n = 249) of the cases in cohort 1, in 4% (n = 17) of the cases in cohort 2, and 13% (n = 40) of the cases in cohort 3.

DISCUSSION

This study explored changes in the outcomes of post-suicide investigations by healthcare services in cases reported as potential incidents of patient harm, adopting a 13-year perspective. Possible

For peer review only - http://bmjopen.bmj.com/site/about/guidelines.xhtml

improvements for patient safety that could contribute to suicide-prevention were also examined in the context of these reports.

Over time the investigations generally and consistently focused on final patient contact, analyzing the immediate interface between the patient and staff from a microsystem level perspective.

The most common measures recommended for all cohorts were updating existing or developing new routines, and educational actions - potentially unsustainable, person-based. Sharing conclusions across departments was planned in only a small percentage of the cases. This similarity of investigation outcomes over the years, regardless of changes in legislation, suggests that the investigations were adapted to suit the structure of the authority report rather than specific incidents, and imply that no new service improvements or lessons are being identified.

The suicide rate in Sweden has not shown any obvious decline since the reporting of all suicide cases became mandatory,[33] and the healthcare-service deficiencies highlighted in these reports as being of significance continue to occur. In other words, despite several thousand investigations into healthcare performance prior to suicides over the last few decades, aimed at identifying actions to improve healthcare for patients with suicidal tendencies, the same contributing factors remain.[34] This suggests that the actions taken to date have not been sufficient. A possible means of addressing this would be the systematic aggregation and analysis of trends through multiple investigations, which would help to conclusively identify recurrent deficiencies, and encouraging investigators to act as facilitators of organizational development instead of mandating single investigations.[35] Another explanation could be that the current investigations fail to identify significant deficiencies, suggesting we need to develop more sophisticated methods for investigations of suicide.

Most of the reported cases in this study had their last contact with a healthcare professional within days of their deaths. Data in this study represent a subset of the total deaths by suicide, excluding these not reported to the authority. However, during the last three years of mandatory reporting (2014-2016), 51-58% of the total suicides in Sweden were reported per year to the supervisory authority.[33 34] Two-thirds of the cases lacked a documented report of an elevated risk of suicide in the months before the death, and this persisted across cohorts, despite the strong focus in many of the analyzed investigations on actions related to suicide-risk assessment and education in this issue. Over the years, there has been a shift from reports of an absence of local policies for suicide-risk assessment to reports of non-adherence to existing policies for suicide-risk assessment. In the studied cohorts, only 7-10% of the patients were documented as being at high risk of suicide during the last months before death. Studies have shown that suicide risk instruments and risk scales do not enable clinicians to predict which patients will die by suicide,[36 37 38] raising the question of the value of these assessments.[39] In an interview study healthcare professionals describe they set forms and checklist aside to prioritise trust during suicide risk assessment.[40]

Approximately half of the suicide victims in all cohorts had a documented prior suicide attempt, and it is shown that previous suicide attempt, especially repeated, imply higher risk for suicide persisting over decades.[41] Learning from cases of the successful treatment of patients who have survived prior suicidal crises could thus be of importance for improving suicide prevention in healthcare. However, such learning actions are not recommended in the Swedish reporting system, which is currently based on a safety-I model; thus possible learning opportunities are not supported unless a safety-II perspective is supplemented.[3]

Cohort 3 showed a higher proportion of deficiencies in “education and competence” when compared to cohorts 1 and 2. These deficiencies were often connected to deficiencies in “human resources” and “internal communication,” suggesting difficulties in recruiting personnel with adequate competence, shortcomings in the introduction of new staff, and complications integrating locum doctors.

For peer review only

Deficiencies in "external communication" and "treatment" were present in almost one-third of the cases in cohort 3. This cohort showed a younger population with some higher degree of psychiatric diagnoses, which suggests that this was a more complex group with a need for support from different care providers, requiring external collaboration and, possibly, more complex treatment interventions.

In all cohorts, there was a pronounced focus on routines. Updating existing or developing new routines was the most common recommendation proposed in the investigations over the years. All cohorts, but most obviously cohort 3, showed a mismatch between the number of cases where an absence of routines was noted and the number of cases for which the development of new routines was recommended. Further, the number of revisions exceeded the number of identified dysfunctional routines. Non-adherence to existing routines was highlighted in almost one-third of the cases in cohort 3, and the solutions seemed to focus on creating new routines instead of ensuring adherence, preconditions, and usability. Notably, reflections on why adherence to existing routines failed from a system perspective were missing in the investigations. This obsession with routines reflects the current predominant perspectives of safety-I. In the perspective of safety-II, the variability of performance conditions that is the reality in healthcare, requires that how the work is performed has to be adopted to the current specific situation to maintain safety.[3 4] Thereby, no precise detailed descriptions of how all work should be done in all situations is possible or even desirable.

Further, changes or reimplementation of routines are person-based and have weak efficacy from a systemic perspective, but require less effort than strong actions on a systemic level.[42 43] The same concerns were present regarding educational actions, which were highlighted in over half of the cases in cohorts 2 and 3. The dominance of person-based actions at the microsystem level is not unique for the Swedish setting. Kellogg et al obtained the same findings in a review conducted in the US,[12] and other studies have reported that investigators complete their analyses after identifying human error, rather than proceeding to identify system-based problems.[44 45] Attributing issues to human error easily leads to person-based solutions, and creates a focus on what is possible rather than what is needed.[35] Recurrent widespread microsystem issues require whole-system responses at macro level to be solved.

Suicide locations and methods were similar in all cohorts, but were reported in less than 90% of the investigations in cohort 3. This was surprising, as these cases were regarded as representing incidents of severe patient harm, and analysis of the specific circumstances concerning the suicide should be of importance in regard to evaluating preventable factors.

The distribution of the supervisory authority's decisions remained similar over the years; most reports were approved without further arrangements. In a small number of cases, the authority made a site visit, but the frequency of such visits declined as time passed. Supervision can be a strong tool and incitement for improvement and development of healthcare services,[14] but the results in this study suggest that the authority did not avail of this. Mandatory reporting thus was determined to be a process of information transfer between healthcare providers and the authority, rather than a means of creating a participative improvement that enhances safety for patients with suicidal tendencies.

The overall aim of the incident-reporting system is to make healthcare safer, which presupposes learning. However, learning that extends beyond the staff involved in the incident requires information-sharing. The review of the reports in this study showed that sharing information between departments was planned in a low percentage of cases, which is in concordance with similar results reported in a previous Swedish study.[10] Learning is a complex social and participative process that involves people actively reflecting on and organizing shared knowledge and practices.[8] Safety begins, rather than ends, with incident reports, and requires broad, in-depth, and high-quality investigations and careful planning and follow-up of the implementation of corrective actions to ensure they are sustainable over time.[46] To generate persistent knowledge and learning from cases, feedback should

For peer review only - http://bmjopen.bmj.com/site/about/guidelines.xhtml

include more than a passive, brief report in a staff meeting that reminds of or notifies of the updating of a routine.

Suicide is usually the final outcome of several interacting factors over time, and only a small proportion of suicides are committed in hospitals.[47 48] Most suicides occur in the patient's home without any witnesses or staff; this makes suicide, as a case of patient harm, somewhat different from most other kinds of such incidents in healthcare. The requirements of the report to the authority are the same for all kinds of incidents, meaning the investigating process may be adapted to suit the standard template rather than the specific character of the incident. Analyzing the last contact with a healthcare professional from a microsystem level perspective is not sufficient to learn how healthcare can better help patients with suicidal tendencies. The investigation should integrate analysis of the suicidal process over time, including suicide-prevention tools. To advance this issue, a shift in investigations requirements and reports is needed, as well as more sophisticated infrastructures for investigation, learning, and sharing in healthcare services. Innovation based on relevant patient safety paradigms combined with suicide preventions research is needed.

Limitations and strengths

All data were based on the healthcare providers' investigations and reports to the supervisory authority, a subset of the total deaths by suicide, excluding these not reported to the authority. The content in the reports is regulated by law; however, the quality of analysis differs and there still may have been additional shortcomings and inadequacies that were not mentioned in the reports or observed by the authority, as well as there were actions mentioned which had no relevance in the circumstances described. Furthermore, there is no national taxonomy for the categorization of deficiencies and actions; a coding scheme created by the authors and used in a prior study was used. The category of "other" was used only in a few cases, suggesting that the categories in the coding scheme covered most of the reported deficiencies and actions.

The strengths of this study are that all investigations concerned the same kind of incident; suicides, and the data were population-based. Further, all data collection and categorization were conducted by only one researcher, who is a psychiatrist with experience working with patient safety issues; this made the categorization vulnerable to bias, but ensured a high level of consistency.

Conclusions

The mandatory reporting of suicides as potential cases of patient harm was shown to be restricted to information transfer between healthcare providers and the supervisory authority, rather than fostering participative improvement of patient safety for suicidal patients.

The similarity in outcomes across the cohorts, regardless of changes in legislation, suggests that the investigations were adapted to suit the structure of the authority's reports rather than the specific incident type, and that no new service improvements or lessons are being identified.

To develop more sophisticated infrastructures for investigation, learning, and information-sharing, it is necessary to learn more about preconditions and complexity in the analysis of suicides and the suicidal process.

A shift in investigations' recommendations and reports should be encouraged, to also include learning from successfully treated and resolved suicide-related crises.

Acknowledgements

The authors are grateful to Region Jönköpings county and Futurum for funding and to Public Health Agency of Sweden for support.

For peer review only - http://bmjopen.bmj.com/site/about/guidelines.xhtml

Authors´ contributor statement

EF designed the study, collected and registered the data, made the first analyses and wrote the manuscript. BAG, AR and ÅW contributed to the study design, analyses of the data and revisions of the manuscript. All authors read and approved the final manuscript.

Competing interests

The authors declare that they have no competing interests.

Funding

This study was funded by Futurum, the research center at Region Jönköping county.

Data sharing statement

The complete coding scheme is available by e-mailing elin.froding@rjl.se.

For peer review only

REFERENCES

1 Kohn LT CJ, Donaldson MS. To err is human: building a safer health system. Wahington DC: National Academies Press 2000.
2 Leape LL. Reporting of adverse events. *N Engl J Med* 2002;347(20):1633-8. doi: 10.1056/NEJMNEJMhpr011493
3 Hollnagel E, Wears RL, Braithwaite J. From Safety-I to Safety-II: a white paper. *Published simultaneously by the University of Southern Denmark, University of Florida, USA, and Macquarie University, Australia: The Resilient Helath Care Net* 2015
4 Braithwaite J, Wears RL, Hollnagel E. Resilient health care: turning patient safety on its head. *Int J Qual Health Care* 2015;27(5):418-20. doi: 10.1093/intqhc/mzv063
5 Stanhope N, Crowley-Murphy M, Vincent C*, et al.* An evaluation of adverse incident reporting. *J Eval Clin Pract* 1999;5(1):5-12. doi: 10.1046/j.1365-2753.1999.00146.x
6 Vincent C, Amalberti R. Safety in healthcare is a moving target. *BMJ Qual Saf* 2015;24(9):539-40. doi: 10.1136/bmjqs-2015-004403
7 Macrae C, Vincent C. Learning from failure: the need for independent safety investigation in healthcare. *J R Soc Med* 2014;107(11):439-43. doi: 10.1177/0141076814555939
8 Macrae C. The problem with incident reporting. *BMJ Qual Saf* 2016;25(2):71-5. doi: 10.1136/bmjqs-2015-004732
9 Macrae C. Remembering to learn: the overlooked role of remembrance in safety improvement. *BMJ Qual Saf* 2017;26(8):678-82. doi: 10.1136/bmjqs-2016-005547
10 Wrigstad J, Bergström J, Gustafsson P. Mind the gap between recommendation and implementation—principles and lessons in the aftermath of incident investigations: a semi-quantitative and qualitative study of factors leading to the successful implementation of recommendations. *BMJ Open* 2014;4 doi: 10.1136/bmjopen-2014-005326
11 Roos af Hjelmsäter E, Ros A, Gäre BA*, et al.* Deficiencies in healthcare prior to suicide and actions to deal with them: a retrospective study of investigations after suicide in Swedish healthcare. *BMJ Open* 2019;9(12):e032290. doi: 10.1136/bmjopen-2019-032290
12 Kellogg KM, Hettinger Z, Shah M*, et al.* Our current approach to root cause analysis: is it contributing to our failure to improve patient safety? *BMJ Qual Saf* 2017;26(5):381-87. doi: 10.1136/bmjqs-2016-005991

For peer review only - http://bmjopen.bmj.com/site/about/guidelines.xhtml

13 Mitchell I, Schuster A, Smith K, *et al.* Patient safety incident reporting: a qualitative study of thoughts and perceptions of experts 15 years after 'To Err is Human'. *BMJ Qual Saf* 2016;25(2):92-9. doi: 10.1136/bmjqs-2015-004405
14 Leistikow I, Mulder S, Vesseur J, *et al.* Learning from incidents in healthcare: the journey, not the arrival, matters. *BMJ Qual Saf* 2017;26(3):252-56. doi: 10.1136/bmjqs-2015-004853
15 Shojania KG, Thomas EJ. Trends in adverse events over time: why are we not improving? *BMJ Qual Saf* 2013;22(4):273-77. doi: 10.1136/bmjqs-2013-001935
16 The Swedish Patient Safety Act (SFS 2010:659) [https://www.riksdagen.se/sv/dokument-lagar/dokument/svensk-forfattningssamling/patientsakerhetslag-2010659_sfs-2010-659]. [accessed 2 February 2019].
17 World Health Organization. Suicide data 2018 [Available from: http://www.who.int/mental_health/prevention/suicide/suicideprevent/en/ [accessed 17 October 2018].
18 Mann J, Currier D. Stress, genetics and epigenetic effects on the neurobiology of suicidal behavior and depression. *European Psychiatry* 2010;25(5):268-71.
19 Luoma JB, Martin CE, Pearson JL. Contact with mental health and primary care providers before suicide: a review of the evidence. *Am J Psychiatry* 2002;159(6):909-16. doi: 10.1176/appi.ajp.159.6.909
20 Chock MM, Lin JC, Athyal VP, *et al.* Differences in health care utilization in the year before suicide death: a population-based case-control ctudy. *Mayo Clin Proc* 2019;94(10):1983-93. doi: 10.1016/j.mayocp.2019.04.037
21 Cavanagh JT, Carson AJ, Sharpe M, *et al.* Psychological autopsy studies of suicide: a systematic review. *Psychol Med* 2003;33(3):395-405.
22 Arsenault-Lapierre G, Kim C, Turecki G. Psychiatric diagnoses in 3275 suicides: a meta-analysis. *BMC psychiatry* 2004;4(1):37.
23 Bertolote JM, Fleischmann A. Suicide and psychiatric diagnosis: a worldwide perspective. *World psychiatry* 2002;1(3):181.
24 Kapur N. Health services and suicide prevention. *Journal of Mental Health* 2009;18(1):1-5. doi: 10.1080/09638230802370704
25 Quinlivan L, Littlewood DL, Webb RT, *et al.* Patient safety and suicide prevention in mental health services: time for a new paradigm? *Journal of Mental Health* 2020;29(1):1-5. doi: 10.1080/09638237.2020.1714013
26 Pirkola S, Sund R, Sailas E, *et al.* Community mental-health services and suicide rate in Finland: a nationwide small-area analysis. *The Lancet* 2009;373(9658):147-53.
27 While D, Bickley H, Roscoe A, *et al.* Implementation of mental health service recommendations in England and Wales and suicide rates, 1997–2006: a cross-sectional and before-and-after observational study. *The Lancet* 2012;379(9820):1005-12.
28 Kapur N, Ibrahim S, While D, *et al.* Mental health service changes, organisational factors, and patient suicide in England in 1997–2012: a before-and-after study. *The Lancet Psychiatry* 2016;3(6):526-34.
29 Socialstyrelsens föreskrifter om anmälan av vårdskador enligt lex Maria (SOSFS 2005:28). Stockholm: The Swedish National Board of Health and Welfare.
30 Socialstyrelsens föreskrifter och allmänna råd om vårdgivares systematiska patientsäkerhetsarbete (HSLF-FS 2017:40). Stockholm: The Swedish National Board of Health and Welfare.
31 Swedish association of local authorities and regions. Risk och händelseanalys [Risk analysis and adverse events analysis]. Stockholm: Swedish association of local authorities and regions 2015.
32 Nelson EC, Batalden PB, Godfrey MM. Quality by design: a clinical microsystems approach. San Fransisco: Jossey-Bass 2007.

For peer review only - http://bmjopen.bmj.com/site/about/guidelines.xhtml

33 The national board of health and welfare. Statistikdatabas för dödsorsaker [Statistical databease, cause of death]: The national board of health and welfare; 2020 [Available from: https://sdb.socialstyrelsen.se/if_dor/resultat.aspx [accessed 2020-02-02.
34 Health and Social Care Inspectorate. https://www.ivo.se/publicerat-material/statistik/lex-maria-och-lex-sarah/ [[accessed 2020-02-05.
35 Nicolini D, Waring J, Mengis J. Policy and practice in the use of root cause analysis to investigate clinical adverse events: mind the gap. *Soc Sci Med* 2011;73(2):217-25. doi: 10.1016/j.socscimed.2011.05.010
36 Runeson B, Odeberg J, Pettersson A*, et al.* Instruments for the assessment of suicide risk: a systematic review evaluating the certainty of the evidence. *PLoS one* 2017;12(7):e0180292.
37 Carter G, Milner A, McGill K*, et al.* Predicting suicidal behaviours using clinical instruments: systematic review and meta-analysis of positive predictive values for risk scales. *The British Journal of Psychiatry* 2017;210(6):387-95.
38 Large M, Smith G, Sharma S*, et al.* Systematic review and meta-analysis of the clinical factors associated with the suicide of psychiatric in-patients. *Acta Psychiatrica Scandinavica* 2011;124(1):18-19.
39 Suicide NCIi, Health SiM. The assessment of clinical risk in mental health services: The University of Manchester Manchester, 2018.
40 Berg SH, Rørtveit K, Walby FA*, et al.* Adaptive capacities for safe clinical practice for patients hospitalised during a suicidal crisis: a qualitative study. *BMC psychiatry* 2020;20(1):1-12.
41 Probert-Lindström S, Berge J, Westrin Å*, et al.* Long-term risk factors for suicide in suicide attempters examined at a medical emergency in patient unit: results from a 32-year follow-up study. *BMJ open* 2020;10(10):e038794.
42 Trbovich P, Shojania KG. Root-cause analysis: swatting at mosquitoes versus draining the swamp. *BMJ Qual Saf* 2017;26(5):350-53. doi: 10.1136/bmjqs-2016-006229
43 Cafazzo JA, St-Cyr O. From discovery to design: the evolution of human factors in healthcare. *Healthc Q* 2012;15(April (Special Issue)):24-29.
44 Mills PD, Neily J, Luan D*, et al.* Actions and implementation strategies to reduce suicidal events in the Veterans Health Administration. *Jt Comm J Qual Patient Saf* 2006;32(3):130-41.
45 Percarpio KB, Watts BV, Weeks WB. The effectiveness of root cause analysis: what does the literature tell us? *Jt Comm J Qual Patient Saf* 2008;34(7):391-8. doi: 10.1016/s1553-7250(08)34049-5
46 Macrae C. Close calls: managing risk and resilience in airline flight safety: Springer 2014.
47 Qin P, Nordentoft M. Suicide risk in relation to psychiatric hospitalization: evidence based on longitudinal registers. *Arch Gen Psychiatry* 2005;62(4):427-32. doi: 10.1001/archpsyc.62.4.427
48 Ballard ED PM, Henderson D, et al. Suicide in the medical setting. *Jt Comm J Qual Patient Saf* 2008;34:474-81.

For peer review only - http://bmjopen.bmj.com/site/about/guidelines.xhtml

STROBE 2007 (v4) Statement—Checklist of items that should be included in reports of *cohort studies*

Section/Topic	Item #	Recommendation	Reported on page #
**Title and abstract**	1	(*a*) Indicate the study's design with a commonly used term in the title or the abstract	1
		(*b*) Provide in the abstract an informative and balanced summary of what was done and what was found	2
**Introduction**			
Background/rationale	2	Explain the scientific background and rationale for the investigation being reported	3-4
Objectives	3	State specific objectives, including any prespecified hypotheses	4
**Methods**			
Study design	4	Present key elements of study design early in the paper	4-5
Setting	5	Describe the setting, locations, and relevant dates, including periods of recruitment, exposure, follow-up, and data collection	4
Participants	6	(*a*) Give the eligibility criteria, and the sources and methods of selection of participants. Describe methods of follow-up	4
		(*b*) For matched studies, give matching criteria and number of exposed and unexposed	Not relevant
Variables	7	Clearly define all outcomes, exposures, predictors, potential confounders, and effect modifiers. Give diagnostic criteria, if applicable	4-5
Data sources/ measurement	8*	For each variable of interest, give sources of data and details of methods of assessment (measurement). Describe comparability of assessment methods if there is more than one group	4-5
Bias	9	Describe any efforts to address potential sources of bias	13
Study size	10	Explain how the study size was arrived at	4
Quantitative variables	11	Explain how quantitative variables were handled in the analyses. If applicable, describe which groupings were chosen and why	5
Statistical methods	12	(*a*) Describe all statistical methods, including those used to control for confounding	5
		(*b*) Describe any methods used to examine subgroups and interactions	5
		(*c*) Explain how missing data were addressed	Not relevant
		(*d*) If applicable, explain how loss to follow-up was addressed	Not relevant
		(*e*) Describe any sensitivity analyses	Not relevant
**Results**			

For peer review only

Participants	13*	(a) Report numbers of individuals at each stage of study—eg numbers potentially eligible, examined for eligibility, confirmed eligible, included in the study, completing follow-up, and analysed	6
		(b) Give reasons for non-participation at each stage	Not relevant
		(c) Consider use of a flow diagram	Not relevant
Descriptive data	14*	(a) Give characteristics of study participants (eg demographic, clinical, social) and information on exposures and potential confounders	6-7
		(b) Indicate number of participants with missing data for each variable of interest	Not relevant
		(c) Summarise follow-up time (eg, average and total amount)	Not relevant
Outcome data	15*	Report numbers of outcome events or summary measures over time	7-10
Main results	16	(*a*) Give unadjusted estimates and, if applicable, confounder-adjusted estimates and their precision (eg, 95% confidence interval). Make clear which confounders were adjusted for and why they were included	7-10
		(*b*) Report category boundaries when continuous variables were categorized	Not relevant
		(*c*) If relevant, consider translating estimates of relative risk into absolute risk for a meaningful time period	Not relevant
Other analyses	17	Report other analyses done—eg analyses of subgroups and interactions, and sensitivity analyses	Not relevant
**Discussion**			
Key results	18	Summarise key results with reference to study objectives	13
**Limitations**			
Interpretation	20	Give a cautious overall interpretation of results considering objectives, limitations, multiplicity of analyses, results from similar studies, and other relevant evidence	11-13
Generalisability	21	Discuss the generalisability (external validity) of the study results	11-13
**Other information**			
Funding	22	Give the source of funding and the role of the funders for the present study and, if applicable, for the original study on which the present article is based	14

*Give information separately for cases and controls in case-control studies and, if applicable, for exposed and unexposed groups in cohort and cross-sectional studies.

Note: An Explanation and Elaboration article discusses each checklist item and gives methodological background and published examples of transparent reporting. The STROBE checklist is best used in conjunction with this article (freely available on the Web sites of PLoS Medicine at http://www.plosmedicine.org/, Annals of Internal Medicine at http://www.annals.org/, and Epidemiology at http://www.epidem.com/). Information on the STROBE Initiative is available at www.strobe-statement.org.

For peer review only

BMJ Open

Suicide as an incident of severe patient harm – a retrospective cohort study of investigations after suicide in Swedish healthcare in a 13-year perspective

Journal:	*BMJ Open*
Manuscript ID	bmjopen-2020-044068.R2
Article Type:	Original research
Date Submitted by the Author:	17-Feb-2021
Complete List of Authors:	Fröding, Elin; Jönköping University; Region Jönköpings län<br>Gäre, Boel Andersson; Jönköping University; Region Jönköpings län, Futurum<br>Westrin, Åsa; Lund University Department of Clinical Sciences Malmo, Division of Psychiatry, Lund University ; Psychiatry Research Skåne, Office for Psychiatry and Habilitation, Region Skåne<br>Ros, Axel; Jönköping University; Region Jönköpings län
**Primary Subject Heading**:	Health services research
Secondary Subject Heading:	Mental health
Keywords:	Health & safety < HEALTH SERVICES ADMINISTRATION & MANAGEMENT, Quality in health care < HEALTH SERVICES ADMINISTRATION & MANAGEMENT, Risk management < HEALTH SERVICES ADMINISTRATION & MANAGEMENT, Suicide & self-harm < PSYCHIATRY

SCHOLARONE™
Manuscripts

I, the Submitting Author has the right to grant and does grant on behalf of all authors of the Work (as defined in the below author licence), an exclusive licence and/or a non-exclusive licence for contributions from authors who are: i) UK Crown employees; ii) where BMJ has agreed a CC-BY licence shall apply, and/or iii) in accordance with the terms applicable for US Federal Government officers or employees acting as part of their official duties; on a worldwide, perpetual, irrevocable, royalty-free basis to BMJ Publishing Group Ltd ("BMJ") its licensees and where the relevant Journal is co-owned by BMJ to the co-owners of the Journal, to publish the Work in this journal and any other BMJ products and to exploit all rights, as set out in our licence.

The Submitting Author accepts and understands that any supply made under these terms is made by BMJ to the Submitting Author unless you are acting as an employee on behalf of your employer or a postgraduate student of an affiliated institution which is paying any applicable article publishing charge ("APC") for Open Access articles. Where the Submitting Author wishes to make the Work available on an Open Access basis (and intends to pay the relevant APC), the terms of reuse of such Open Access shall be governed by a Creative Commons licence – details of these licences and which Creative Commons licence will apply to this Work are set out in our licence referred to above.

Other than as permitted in any relevant BMJ Author's Self Archiving Policies, I confirm this Work has not been accepted for publication elsewhere, is not being considered for publication elsewhere and does not duplicate material already published. I confirm all authors consent to publication of this Work and authorise the granting of this licence.

For peer review only

Title page

Suicide as an incident of severe patient harm – a retrospective cohort study of investigations after suicide in Swedish healthcare in a 13-year perspective

Authors

PhD. Elin Fröding, Prof. Boel Andersson Gäre, Prof. Åsa Westrin, MD. Axel Ros

Corresponding author

Name and address: Elin Fröding, Höglandssjukhuset Hus 34 pl 4, 575 81 Eksjö, Sweden

Mail and phone number: Elin.froding@rjl.se, +4670-3577396

Co-authors

Prof. Boel Andersson Gäre, Jönköping University, Jönköping, Sweden and Futurum, Region Jönköping County, Sweden.

Prof. Åsa Westrin, Department of Clinical Sciences Lund, Psychiatry, Lund University, Sweden and Office for Psychiatry and Habilitation, Psychiatry Research Skåne, Region Skåne, Sweden.

MD. Axel Ros, Jönköping University, Jönköping, Sweden

Key words

Health & safety, patient safety, risk management, quality in health care, suicide

Word count

4374

For peer review only - http://bmjopen.bmj.com/site/about/guidelines.xhtml

ABSTRACT

Objectives

To explore how mandatory reporting to the supervisory authority of suicides among recipients of healthcare services has influenced associated investigations conducted by the healthcare services, the lessons obtained, and whether any suicide-prevention-related improvements in terms of patient safety had followed.

Design and settings

Retrospective study of reports from Swedish primary and secondary healthcare to the supervisory authority after suicide.

Participants

Cohort 1: the cases reported to the supervisory authority in 2006, from the time the reporting of suicides became mandatory, to 2007 (n = 279). Cohort 2: the cases reported in 2015, a period of well-established reporting (n = 436). Cohort 3: the cases reported from September 2017, which was the time the law regarding reporting was removed, to November 2019 (n = 316).

Primary and secondary outcome measures

Demographic data and received treatment in the months preceding suicide were registered. Reported deficiencies in healthcare and actions were categorized by using a coding scheme, analyzed per individual and aggregated per cohort. Separate notes were made when a deficiency or action was related to a healthcare-service routine.

Results

The investigations largely adopted a microsystem perspective, focusing on final patient contact, throughout the overall study period. Updating existing or developing new routines as well as educational actions were increasingly proposed over time, while sharing conclusions across departments rarely was recommended.

Conclusions

The mandatory reporting of suicides as potential cases of patient harm was shown to be restricted to information transfer between healthcare providers and the supervisory authority, rather than fostering participative improvement of patient safety for suicidal patients.

The similarity in outcomes across the cohorts, regardless of changes in legislation, suggests that the investigations were adapted to suit the structure of the authority’s reports rather than the specific incident type, and that no new service improvements or lessons are being identified.

Strengths and limitations of the study

- To our knowledge, this is the first evaluation of the outcomes of investigations of specific types of patient harm over time, here exemplified by suicide.
- All investigations concerned the same kind of incident; suicides, and the data were population-based.
- All data were based on the healthcare providers’ investigations and reports to the supervisory authority, the content in these reports is regulated by law; however, the quality of analysis differs, which was not evaluated in this study.

For peer review only - http://bmjopen.bmj.com/site/about/guidelines.xhtml

- All data collection and categorization were conducted by only one researcher, which rendered categorization vulnerable to bias; however this ensured a high level of consistency.

BACKGROUND

Deaths that occur as a result of patient harm represent a contrast to healthcare services' aim of a high level of patient safety, and such incidents can serve as powerful motivators for learning and improvement.[1 2] In recent decades, efforts to increase patient safety have been intensified. In particular, the reporting and investigating of cases of severe patient injury in order to identify risks and improve patient safety have become widespread safety-improvement strategies.[2] This reflects a safety-I perspective regarding patient safety, with focus on incidents that could have or did lead to harm for patients during healthcare, assuming that safety is achieved by eliminating what can go wrong.[3] This perspective assumes that adverse outcomes are caused by identifiable failures or malfunctions of specific components different from situations when things go right.[3 4] Similarly, root cause analysis (RCA) has become one of the most widespread tools used in the investigation of healthcare-related incidents, and presumes that such incidents can be explained by linear cause-effect chains.[5 6] Determining what had happened and why an incident occurred should not be the final goal of an incident investigation; the identification of gaps in service provision and means of improving relevant areas of the healthcare organization are important for improving safety.[7] To successfully learn from past incidents, methods to sustainably record and share relevant data are essential.[8 9] However, prior studies have shown that, in healthcare, post-incident investigations usually provide little learning beyond the staff and units involved.[10 11] Thus, the actual value of incident-reporting systems and the RCA approach in healthcare has been questioned.[8 12-15] With the introduction of new concepts in patient safety, such as safety-II and resilient healthcare, new approaches for improving healthcare have focused on learning from all occurrences in daily practice: to identify both those factors that support a good outcome and those that increase the risk of patient harm.[3 4] In the concept of safety-II, focus is on "work in practice", i. e. to better understand how clinicians provide good quality healthcare in real-time dynamic systems, including the interactions between patient care, environmental contexts, and healthcare culture. In this perspective, safety is achieved through understanding health-care staff's adaptations to varying conditions and ensuring that as much as possible goes well.

Swedish law states that events with severe patient harm, as well as events involving risk of severe patient harm, that could have been avoided if appropriate actions had been taken by healthcare professionals, should be reported to the supervisory authority.[16] This report to the authority should be preceded by an investigation, conducted by the healthcare providing organization, of the healthcare services provided to the patient before the adverse event. The content of the investigation is regulated by law, and requires identification of the contributory causes of the incident and of service improvements that may prevent the reoccurrence of such an incident.

Suicide is a global health problem with an estimated 800 000 deaths worldwide every year.[17] Suicidal behaviours are heterogeneous and complex and influenced by several interacting biological, genetic, psychological, social, environmental and situational factors over time.[18] A large proportion of the individuals who die from suicide have contact with healthcare professionals close in time before their deaths.[19 20] Post-suicide studies have found that the vast majority of suicide victims have psychiatric illnesses at the time of their deaths.[21 22 23] This suggests that healthcare professionals play an important role in suicide prevention.[24] However, the nature of suicide as a process going on over time, usually occurring outside the hospitals without any witnesses nor staff around, make suicide as a case of patient harm, somewhat different from most other kinds of such incidents in healthcare. Few studies have applied patient safety paradigms to advance understanding of preventing suicide[25] although there are examples of studies of health services associated with reductions in suicide rates, such as well-developed community outpatient services[26] and the implementation of 24-hour crisis services.[27] Kapur

For peer review only - http://bmjopen.bmj.com/site/about/guidelines.xhtml

et al. suggest system-wide changes implemented across the patient care pathway could be a key strategy for improving patient safety in mental health care.[28]

In an effort to understand whether failures in any area of the healthcare system have contributed to suicide, and in an attempt to improve suicide-prevention, the Swedish National Board of Health and Welfare in 2006 stipulated that all suicides that occur among patients who were receiving healthcare or were in contact with healthcare services within the four weeks preceding the event must be reported to the authority by the healthcare provider.[29] This remained mandatory regardless of whether the provider determined the suicide to be preventable. In September 2017, this regulation was updated to state that only suicides regarded as "severe patient harm" (i.e., preventable) must be reported to the supervisory authority.[30]

Before 2011, the supervisory authority performed their own investigations of incidents, and had the power to reprimand the provider and responsible staff. The role of the supervisory authority changed in 2011, when the Swedish Patient Safety Act (2010:659)[16] was implemented. This new law made healthcare organizations responsible for patient-safety improvement, and the role of the supervisory authority was changed to review the investigations made by the providers, and ensure that they were satisfactorily fulfilled and that appropriate actions had been taken to ensure a high level of patient safety. In particular, the authority determines whether the healthcare provider has fulfilled their legislated duties, or whether there are shortcomings in the investigation, in which case the authority may recommend revisions or conduct a site visit to inspect the healthcare provider.

To our knowledge, there are no published evaluations of the outcomes of investigations of specific types of patient harm over time, here exemplified by suicide.

The objective of this study was to explore how mandatory reporting of suicide cases as incidents of potential patient harm has influenced the investigations of healthcare systems. To perform this, a 13-year perspective was adopted, and the lessons and possible improvements for patient safety regarding suicide prevention were examined.

METHODS

This study followed the guidelines of the STROBE checklist for reporting observational studies, available as a supplementary file.

Cases

Three cohorts of suicide cases, each from a different time period, that were reported to the supervisory authority were chosen for analysis. Cohort 1 comprised the cases reported to the supervisory authority in 2006, from the time the reporting of suicides became mandatory, to 2007 (n = 279). Cohort 2 comprised all suicides reported in 2015, this represented a period when mandatory reporting was well-established among healthcare providers (n = 436). Cohort 3 comprised all reported suicides from September 1, 2017, which was the time the law regarding reporting was changed, to November 30, 2019 (n = 316).

Complete reports of the incident investigations conducted by the healthcare providers with associated patient records and the subsequent decisions of the supervisory authority were obtained from the supervisory authority, granted by a contract of secrecy. Every individual suicide case was given a code number and the patient's demographic data and treatment received in the months preceding his/her death were registered. Major diagnoses were documented and coded in accordance with the International Statistical Classification of Diseases and related Health Problems, 10th revision (i.e., ICD-10).

For peer review only - http://bmjopen.bmj.com/site/about/guidelines.xhtml

Categorization of data

A coding scheme was used to categorize the contributory causes of the respective suicides, the actions reported in the investigations and the decisions of the authority. The same coding scheme was used in a prior study of reported suicide cases in Sweden.[11] This scheme is based on the general categories used in the most common method of investigating adverse events in Swedish healthcare, which is in turn based on RCA.[31] To make the categorization more specific, four of the major categories were divided into additional subcategories. Every category was described and exemplified and a category of "others" was added in case none of the other categories was considered appropriate. In this present study, the contributory causes were reported as "deficiencies." Meanwhile, an "action" was defined as any intervention performed in attempt to prevent new suicides: therefor, actions taken to prevent reported suicides (telephone calls, resuscitations) or actions aimed at informing family members or staff that a suicide had occurred were not registered as actions in this study. Separate notes were made when a deficiency or action was related to a healthcare-service routine, as well as in regard to how learning from the investigation was described. To ensure consistency, all data collection and categorization were conducted by only one researcher (EF), a psychiatrist with extensive experience in patient-safety issues.

Organizational levels

Classification of the organizational levels of deficiencies and actions was conducted to better understand where in the organizational system the identified deficiencies and actions were situated. The deficiencies and actions were coded based on a micro-meso-macro-perspective.[32] Microsystems were defined as the basic elements of the healthcare services provided for the patient, such as the inpatient or outpatient care unit. The mesosystem encompassed interactions between different microsystem units, such as cooperation between departments or different healthcare providers. The macrosystem involved the entire healthcare system, such as legislation, political prioritizations, and national policies on healthcare. For each case, the highest organizational level for each deficiency and action was coded.

Supervisory authority

The mandate stipulated to the authority by legislation differed between cohort 1 and cohorts 2 and 3, hence the formulation of the decisions also differed. In this paper, to facilitate comparison among these outcomes, for all cohorts only decisions categorized as "immediate approval" and "inspection" were noted, as these remained unchanged. A note was made if a physician employed by the supervisory authority was involved in the decision-making.

Statistical analyses

Frequencies for each category, organizational hierarchal level of deficiencies and actions, and decisions of the supervisory authority were analyzed per individual and aggregated per cohort.

Chi-square tests of independence were used to compare the number of new routines and the absence of routines within the same cohort, as well as the proportion of the organizational hierarchy of deficiencies and actions between cohorts. We considered a two-sided p value of < 0.05 to indicate statistical significance. As the pre-requisites differed between the cohorts, no further statistical analyses to compare the cohorts were judged to be possible.

The statistical analyses were performed using IBM SPSS Statistics 24.

Ethical review

For peer review only
For peer review only - http://bmjopen.bmj.com/site/about/guidelines.xhtml

According to the Swedish *Act Concerning the Ethical Review of Research Involving Humans* (2003:460) and an advisory opinion from the Regional Ethical Review Board (no. 2017/234), this study did not require an ethical review as it did not include human participants.

Patient and Public Involvement

Patients or public were not involved in this study.

RESULTS

Cases

Demographic data for the cases showed similarities across the cohorts, with a dominance of men and a majority of cases reported by psychiatric care. One-fourth of the cases died from suicide within one day of their last contact with a healthcare professional; half of the cases died from suicide within 2-4 days of their last contact. For details, see Table 1.

Table 1. Characteristics of the suicide cases reported to the supervisory authority across the three cohorts. The data in the table comprise numbers and percentages, n (%).

		Cohort 1 (n = 279)	Cohort 2 (n = 436)	Cohort 3 (n = 316)
**Characteristic**				
**Age, years**	Range	15-95	13-93	11-95
	Percentile 25	36	33	29
	Percentile 50	50	49	42
	Percentile 75	64	61	57
**Gender**	Men	166 (60)	283 (65)	213 (67)
	Women	113 (40)	152 (35)	103 (33)
**Reporting healthcare service**	Psychiatric care	195 (70)	290 (67)	233 (74)
	Primary care	47 (17)	94 (22)	56 (18)
	Somatic care	21 (7)	33 (8)	16 (5)
	Other	16 (6)	18 (4)	11 (3)
**Days between last contact with healthcare services and death**	Range	0-70	0-88	0-240
	Percentile 25	0	1	0
	Percentile 50	2	4	3
	Percentile 75	7	10	9
**Receiving inpatient care at time of death**		45 (16)	36 (8)	44 (14)
**Receiving compulsory psychiatric treatment at the time of death***		15 (5)	22 (5)	20 (6)
**Major psychiatric diagnosis documented and coded in accordance with ICD-10 in patients' records**	Total (F00-F98)	228 (82)	371 (85)	288 (91)
	Affective disorder (F30)	119 (43)	153 (35)	105 (33)
	Anxiety disorder (F40)	35 (13)	77 (18)	60 (19)
	Substance abuse (F10)	29 (10)	51 (12)	37 (12)
	Psychosis (F20)	22 (8)	36 (8)	30 (10)

For peer review only - http://bmjopen.bmj.com/site/about/guidelines.xhtml

	Personality disorder (F60)	12 (4)	13 (3)	13 (4)
	Attention deficit disorder (F90)	1 (0)	13 (3)	12 (4)
	Autism spectrum (F84)	3 (1)	13 (3)	9 (3)
	Other	7 (2)	15 (3)	22 (7)
**Suicide-risk assessment documented in patients' records in the three months before death**	Absent	135 (49)	108 (25)	119 (38)
	Low	61 (22)	171 (39)	91 (29)
	Elevated, not acute	61 (22)	116 (27)	75 (24)
	High/acute	19 (7)	41 (9)	31 (10)
**Prior suicide attempt**		120 (46)	204 (47)	154 (49)
**Suicide method**	Hanging	112 (40)	160 (37)	128 (41)
	Intoxication	42 (15)	110 (25)	53 (17)
	Jumping	21 (8)	13 (3)	19 (6)
	Train	11 (4)	35 (8)	22 (7)
	Drowning	15 (5)	28 (6)	13 (4)
	Shooting	10 (4)	27 (6)	14 (4)
	Others	13 (8)	12 (3)	16 (5)
	Not reported	51 (18)	50 (12)	51 (16)
**Location of suicide**	Home	154 (56)	248 (57)	161 (51)
	Hospital	23 (8)	22 (5)	33 (10)
	Other	53 (19)	131 (30)	83 (26)
	Not reported	44 (16)	35 (8)	39 (12)

Note: Cohort 1: cases reported in 2006-2007, cohort 2: cases reported in 2015, and cohort 3: cases reported in 2017-2019. ICD-10: International Classification of Diseases and related Health Problems, 10th revision.

* includes both in-patient and out-patient compulsory treatment

Deficiencies in healthcare

Cohort 3 showed the largest proportion of cases for which deficiencies in healthcare were considered to have contributed to the suicide. In this cohort, only suicide cases considered to involve severe patient harm could have been prevented if different actions had been taken by healthcare professionals were to be reported. Over time, some changes in the proportions for the categories of deficiencies were observed, but they remained centered on final patient contact with healthcare services. In cohort 1 and 2, the most common deficiencies concerned "suicide risk assessment." In general, in cohort 1 these deficiencies related to an absence of local guidelines for suicide risk assessment, and in cohort 2 to non-adherence to existing guidelines. In cohort 3, deficiencies in "treatment" and "external communication" were the most common. Examples of deficiencies in "treatment" were delayed, or a lack of, follow-up after prescription of medication, or non-adherence to treatment guidelines. Examples of deficiencies in "external information" were a lack of or insufficient information exchange between healthcare providers. For details, see Table 2.

For peer review only - http://bmjopen.bmj.com/site/about/guidelines.xhtml

Table 2. Proportions of cases with deficiencies, as reported in the post-suicide investigations of the healthcare services' actions. The data in the table comprise numbers and percentages, n (%).

	Cohort 1 (n = 279)	Cohort 2 (n = 436)	Cohort 3 (n = 316)
**Cases with deficiencies, total**	136 (49)	240 (55)	248 (78)
**Category**			
**Communication and information**			
Communication with peers and family members	8 (3)	51 (12)	39 (12)
Documentation	57 (20)	65 (15)	68 (22)
External communication	21 (8)	74 (17)	91 (29)
Internal communication	18 (7)	61 (14)	68 (22)
**Education and competence**			
Education and competence not specified	12 (4)	54 (11)	50 (16)
Education and competence in suicide risk assessment	5 (2)	9 (2)	13 (4)
**Organization and management**			
Human resources	15 (5)	60 (14)	53 (17)
Number of beds	2 (1)	9 (2)	5 (2)
Organization/management	2 (1)	13 (3)	13 (4)
**Policies and procedures**			
Treatment	26 (9)	84 (19)	92 (29)
Suicide risk assessment	92 (33)	86 (20)	76 (24)
Work process	20 (7)	50 (11)	51 (16)
Diagnostics	16 (6)	54 (12)	41 (13)
Care plan and crisis plan	10 (4)	46 (11)	53 (17)
**Technics and equipment**	5 (2)	13 (3)	15 (5)
**Other**	2 (1)	11 (3)	0 (0)

Note. Cohort 1: cases reported in 2006-2007, cohort 2: cases reported in 2015, and cohort 3: cases reported in 2017-2019.

Proposed actions for addressing deficiencies

In a majority of the cases, the providers proposed actions for improving the healthcare services. The proportions of the action categories differed between the cohorts. In cohort 1, actions relating to "suicide risk assessment" were most common, usually involving the creation of new local guidelines regarding this issue. In cohorts 2 and 3, actions centered on education, present in more than half of the cases. Examples of educational actions were reminding staff about existing local guidelines, holding case-report discussions at staff meetings, and staging lectures regarding suicide risk assessment. For details, see Table 3.

Table 3. Proportions of cases for which actions were recommended in the post-suicide investigations. The data in the table comprise numbers and percentages, n (%).

	Cohort 1 (n = 279)	Cohort 2 (n =436)	Cohort 3 (n = 316)

For peer review only - http://bmjopen.bmj.com/site/about/guidelines.xhtml

**Cases with actions, total**	133 (48)	346 (79)	283 (90)
**Category**			
**Communication and information**			
Communication with peers and family	12 (4)	51 (12)	27 (9)
Documentation	39 (14)	71 (16)	65 (21)
External communication	22 (8)	80 (18)	83 (26)
Internal communication	15 (5)	55 (13)	46 (15)
**Education and competence**			
Education and competence not specified	35 (13)	166 (38)	136 (43)
Education and competence in suicide risk assessment	44 (16)	136 (31)	85 (27)
**Organization and management**			
Human resources	7 (3)	67 (15)	42 (13)
Number of beds	1 (0)	4 (1)	1 (0)
Organization/management	6 (2)	22 (5)	20 (6)
**Policies and procedures**			
Treatment	21 (8)	56 (13)	64 (20)
Suicide risk assessment	74 (27)	94 (22)	51 (16)
Work process	28 (10)	119 (27)	87 (28)
Diagnostics	8 (3)	28 (6)	25 (8)
Care plan and crisis plan	6 (2)	46 (11)	51 (16)
**Technics and equipment**	12 (4)	22 (5)	22 (7)
**Other**	1 (0)	8 (2)	3 (1)

Note. Cohort 1 comprises of cases reported in 2006-2007, cohort 2 cases reported in 2015, and cohort 3 cases reported in 2017-2019.

Learning and sharing

Any lessons learned and the sharing of experiences obtained from cases and investigations usually remained within the department in question. Sharing outside the department was reported in 4% (n = 17) of the cases in cohort 2, and in 7% (n = 21) of the cases in cohort 3. Sharing outside the department was not reported in any cases in cohort 1.

Routines

Over time, proposals for actions concerning updating or developing new routines became more common in the investigations. In cohorts 2 and 3, there were significantly more cases featuring the proposed development of new routines when compared with the number of cases for which an absence of routines was identified. In all cohorts, the number of revisions exceeded the number of identified dysfunctional routines. Non-adherence to existing routines was highlighted in almost one-third of the cases in cohort 3. For details, see Table 4.

Table 4. Deficiencies and actions in routines, reported in the post-suicide investigations.

		**Cohort 1 (n=279)**	**Cohort 2 (n=436)**	**Cohort 3 (n=316)**
**Routines, deficiencies**	Non-adherence	10 (4)	44 (10)	95 (30)

For peer review only - http://bmjopen.bmj.com/site/about/guidelines.xhtml

	Absent	38 (14)	30 (7)	28 (9)
	Dysfunctional	1 (0)	0 (0)	8 (3)
**Routines, actions**	Revision	24 (9)	58 (13)	47 (15)
	New	55 (20)	94 (22)*	99 (31)*

Note. The data in the table comprise numbers and percentage, n (%). Cohort 1: cases reported in 2006-2007, cohort 2: cases reported in 2015, and cohort 3: cases reported in 2017-2019.

* Significantly more cases involved the development of new routines when compared with the number of absent routines, p < 0.001

Organizational hierarchy

For both deficiencies and proposed actions, the microsystem perspective remained dominant over the 13-year period. However, cohorts 2 and 3 showed a significant increase in the proportion of deficiencies and actions at the mesosystem level compared with cohort 1. No deficiencies were found at the macrosystem level. For details, see Table 5.

Examples of deficiencies at the microsystem level were inadequacies in doctors' prescriptions or in suicide-risk assessments. Examples of actions at the microsystem level were case discussions at staff meetings, lectures, and the development of new checklists. Deficiencies at the mesosystem level included shortcomings in cooperation between the psychiatric clinic and somatic clinic, or inadequate communication between the hospital and primary care center. Examples of actions at the mesosystem level were alterations of procedures for communication or cooperation between different healthcare providers.

Table 5. Respective distributions of the highest organizational hierarchy levels for the deficiencies and actions associated with the cases. Only the highest level for each case is noted. The data in the table comprise numbers and percentages, n (%).

		Cohort 1	Cohort 2	Cohort 3
Organizational level, deficiencies	**Micro**	121 (90)	157 (65)	179 (73)
	**Meso**	13 (10)	83 (35)*	67 (27)*
	**Macro**	0 (0)	0 (0)	0 (0)
Organizational level, actions	**Micro**	115 (85)	225 (65)	206 (75)
	**Meso**	20 (15)	120 (35)*	70 (25)*
	**Macro**	0 (0)	1 (0)	0 (0)

Note. Cohort 1: cases reported in 2006-2007, cohort 2: cases reported in 2015, and cohort 3: cases reported in 2017-2019.

* Significantly larger proportion of cases with deficiencies or actions at the mesosystem level when compared to cohort 1, p < 0.005

Decisions of the supervisory authority

In all cohorts, the majority of the reports from the healthcare providers were approved by the supervisory authority without further requirements. Immediate approval was provided for 59% (n = 164) of the reports for cohort 1, 65% (n = 284) for cohort 2, and 59% (n = 186) for cohort 3. Meanwhile, inspections of the healthcare provider occurred for 9% (n = 25) of the cases in cohort 1, 6% (n = 25) of those in cohort 2 and 4% (n = 13) of those in cohort 3. A physician employed at the supervisory authority was involved in the decision-making for 89% (n = 249) of the cases in cohort 1, in 4% (n = 17) of the cases in cohort 2, and 13% (n = 40) of the cases in cohort 3.

For peer review only - http://bmjopen.bmj.com/site/about/guidelines.xhtml

DISCUSSION

This study explored changes in the outcomes of post-suicide investigations by healthcare services in cases reported as potential incidents of patient harm, adopting a 13-year perspective. Possible improvements for patient safety that could contribute to suicide-prevention were also examined in the context of these reports.

Over time the investigations generally and consistently focused on final patient contact, analyzing the immediate interface between the patient and staff from a microsystem level perspective.

The most common measures recommended for all cohorts were updating existing or developing new routines, and educational actions - potentially unsustainable, person-based. Sharing conclusions across departments was planned in only a small percentage of the cases. This similarity of investigation outcomes over the years, regardless of changes in legislation, suggests that the investigations were adapted to suit the structure of the authority report rather than specific incidents, and imply that no new service improvements or lessons are being identified.

The suicide rate in Sweden has not shown any obvious decline since the reporting of all suicide cases became mandatory,[33] and the healthcare-service deficiencies highlighted in these reports as being of significance continue to occur. In other words, despite several thousand investigations into healthcare performance prior to suicides over the last few decades, aimed at identifying actions to improve healthcare for patients with suicidal tendencies, the same contributing factors remain.[34] This suggests that the actions taken to date have not been sufficient. A possible means of addressing this would be the systematic aggregation and analysis of trends through multiple investigations, which would help to conclusively identify recurrent deficiencies, and encouraging investigators to act as facilitators of organizational development instead of mandating single investigations.[35] Another explanation could be that the current investigations fail to identify significant deficiencies, suggesting we need to develop more sophisticated methods for investigations of suicide.

Most of the reported cases in this study had their last contact with a healthcare professional within days of their deaths. Data in this study represent a subset of the total deaths by suicide, excluding these not reported to the authority. However, during the last three years of mandatory reporting (2014-2016), 51-58% of the total suicides in Sweden were reported per year to the supervisory authority.[33 34] Two-thirds of the cases lacked a documented report of an elevated risk of suicide in the months before the death, and this persisted across cohorts, despite the strong focus in many of the analyzed investigations on actions related to suicide-risk assessment and education in this issue. Over the years, there has been a shift from reports of an absence of local policies for suicide-risk assessment to reports of non-adherence to existing policies for suicide-risk assessment. In the studied cohorts, only 7-10% of the patients were documented as being at high risk of suicide during the last months before death. Studies have shown that suicide risk instruments and risk scales do not enable clinicians to predict which patients will die by suicide, [36 37 38] raising the question of the value of these assessments.[39] In an interview study healthcare professionals describe they set forms and checklist aside to prioritise trust during suicide risk assessment.[40]

Approximately half of the suicide victims in all cohorts had a documented prior suicide attempt, and it is shown that previous suicide attempt, especially repeated, imply higher risk for suicide persisting over decades. [41] Learning from cases of the successful treatment of patients who have survived prior suicidal crises could thus be of importance for improving suicide prevention in healthcare. However, such learning actions are not recommended in the Swedish reporting system, which is currently based on a safety-I model; thus possible learning opportunities are not supported unless a safety-II perspective is supplemented.[3]

Cohort 3 showed a higher proportion of deficiencies in “education and competence” when compared to cohorts 1 and 2. These deficiencies were often connected to deficiencies in “human resources” and

For peer review only - http://bmjopen.bmj.com/site/about/guidelines.xhtml

"internal communication," suggesting difficulties in recruiting personnel with adequate competence, shortcomings in the introduction of new staff, and complications integrating locum doctors.

Deficiencies in "external communication" and "treatment" were present in almost one-third of the cases in cohort 3. This cohort showed a younger population with some higher degree of psychiatric diagnoses, which suggests that this was a more complex group with a need for support from different care providers, requiring external collaboration and, possibly, more complex treatment interventions.

In all cohorts, there was a pronounced focus on routines. Updating existing or developing new routines was the most common recommendation proposed in the investigations over the years. All cohorts, but most obviously cohort 3, showed a mismatch between the number of cases where an absence of routines was noted and the number of cases for which the development of new routines was recommended. Further, the number of revisions exceeded the number of identified dysfunctional routines. Non-adherence to existing routines was highlighted in almost one-third of the cases in cohort 3, and the solutions seemed to focus on creating new routines instead of ensuring adherence, preconditions, and usability. Notably, reflections on why adherence to existing routines failed from a system perspective were missing in the investigations. This obsession with routines reflects the current predominant perspectives of safety-I. In the perspective of safety-II, the variability of performance conditions that is the reality in healthcare, requires that how the work is performed has to be adopted to the current specific situation to maintain safety.[3 4] Thereby, no precise detailed descriptions of how all work should be done in all situations is possible or even desirable.

Further, changes or reimplementation of routines are person-based and have weak efficacy from a systemic perspective, but require less effort than strong actions on a systemic level.[42 43] The same concerns were present regarding educational actions, which were highlighted in over half of the cases in cohorts 2 and 3. The dominance of person-based actions at the microsystem level is not unique for the Swedish setting. Kellogg et al obtained the same findings in a review conducted in the US,[12] and other studies have reported that investigators complete their analyses after identifying human error, rather than proceeding to identify system-based problems.[44 45] Attributing issues to human error easily leads to person-based solutions, and creates a focus on what is possible rather than what is needed.[35] Recurrent widespread microsystem issues require whole-system responses at macro level to be solved.

Suicide locations and methods were similar in all cohorts, but were reported in less than 90% of the investigations in cohort 3. This was surprising, as these cases were regarded as representing incidents of severe patient harm, and analysis of the specific circumstances concerning the suicide should be of importance in regard to evaluating preventable factors.

The distribution of the supervisory authority's decisions remained similar over the years; most reports were approved without further arrangements. In a small number of cases, the authority made a site visit, but the frequency of such visits declined as time passed. Supervision can be a strong tool and incitement for improvement and development of healthcare services,[14] but the results in this study suggest that the authority did not avail of this. Mandatory reporting thus was determined to be a process of information transfer between healthcare providers and the authority, rather than a means of creating a participative improvement that enhances safety for patients with suicidal tendencies.

The overall aim of the incident-reporting system is to make healthcare safer, which presupposes learning. However, learning that extends beyond the staff involved in the incident requires information-sharing. The review of the reports in this study showed that sharing information between departments was planned in a low percentage of cases, which is in concordance with similar results reported in a previous Swedish study.[10] Learning is a complex social and participative process that involves people actively reflecting on and organizing shared knowledge and practices.[8] Safety begins, rather than ends, with incident reports, and requires broad, in-depth, and high-quality investigations

For peer review only - http://bmjopen.bmj.com/site/about/guidelines.xhtml

and careful planning and follow-up of the implementation of corrective actions to ensure they are sustainable over time.[46] To generate persistent knowledge and learning from cases, feedback should include more than a passive, brief report in a staff meeting that reminds of or notifies of the updating of a routine.

Suicide is usually the final outcome of several interacting factors over time, and only a small proportion of suicides are committed in hospitals.[47 48] Most suicides occur in the patient's home without any witnesses or staff; this makes suicide, as a case of patient harm, somewhat different from most other kinds of such incidents in healthcare. The requirements of the report to the authority are the same for all kinds of incidents, meaning the investigating process may be adapted to suit the standard template rather than the specific character of the incident. Analyzing the last contact with a healthcare professional from a microsystem level perspective is not sufficient to learn how healthcare can better help patients with suicidal tendencies. The investigation should integrate analysis of the suicidal process over time, including suicide-prevention tools. To advance this issue, a shift in investigations requirements and reports is needed, as well as more sophisticated infrastructures for investigation, learning, and sharing in healthcare services. Innovation based on relevant patient safety paradigms combined with suicide preventions research is needed.

Limitations and strengths

All data were based on the healthcare providers' investigations and reports to the supervisory authority, a subset of the total deaths by suicide, excluding these not reported to the authority. The content in the reports is regulated by law; however, the quality of analysis differs and there still may have been additional shortcomings and inadequacies that were not mentioned in the reports or observed by the authority, as well as there were actions mentioned which had no relevance in the circumstances described. Furthermore, there is no national taxonomy for the categorization of deficiencies and actions; a coding scheme created by the authors and used in a prior study was used. The category of "other" was used only in a few cases, suggesting that the categories in the coding scheme covered most of the reported deficiencies and actions.

The strengths of this study are that all investigations concerned the same kind of incident; suicides, and the data were population-based. Further, all data collection and categorization were conducted by only one researcher, who is a psychiatrist with experience working with patient safety issues; this made the categorization vulnerable to bias, but ensured a high level of consistency.

Conclusions

The mandatory reporting of suicides as potential cases of patient harm was shown to be restricted to information transfer between healthcare providers and the supervisory authority, rather than fostering participative improvement of patient safety for suicidal patients.

The similarity in outcomes across the cohorts, regardless of changes in legislation, suggests that the investigations were adapted to suit the structure of the authority's reports rather than the specific incident type, and that no new service improvements or lessons are being identified.

To develop more sophisticated infrastructures for investigation, learning, and information-sharing, it is necessary to learn more about preconditions and complexity in the analysis of suicides and the suicidal process.

A shift in investigations' recommendations and reports should be encouraged, to also include learning from successfully treated and resolved suicide-related crises.

Acknowledgements

For peer review only - http://bmjopen.bmj.com/site/about/guidelines.xhtml

The authors are grateful to Region Jönköpings county and Futurum for funding and to Public Health Agency of Sweden for support.

Authors´ contributor statement

EF designed the study, collected and registered the data, made the first analyses and wrote the manuscript. BAG, AR and ÅW contributed to the study design, analyses of the data and revisions of the manuscript. All authors read and approved the final manuscript.

Competing interests

The authors declare that they have no competing interests.

Funding

This study was funded by Futurum, the research center at Region Jönköping county.

Data sharing statement

The complete coding scheme is available by e-mailing elin.froding@rjl.se.

The data of the case reports were obtained from the supervisory authorities, granted by a contract of secrecy. The data can only be obtained by direct request to the supervisory authorities: for cohort 1: The national board of health and welfare, mail: Registerservice@socialstyrelsen.se, and for cohort 2 and 3: Health and Social Care Inspectorate, mail: samordna.utlamnanden@ivo.se.

REFERENCES

1 Kohn LT CJ, Donaldson MS. To err is human: building a safer health system. Wahington DC: National Academies Press 2000.

2 Leape LL. Reporting of adverse events. *N Engl J Med* 2002;347(20):1633-8. doi: 10.1056/NEJMNEJMhpr011493

3 Hollnagel E, Wears RL, Braithwaite J. From Safety-I to Safety-II: a white paper. *Published simultaneously by the University of Southern Denmark, University of Florida, USA, and Macquarie University, Australia: The Resilient Helath Care Net* 2015

4 Braithwaite J, Wears RL, Hollnagel E. Resilient health care: turning patient safety on its head. *Int J Qual Health Care* 2015;27(5):418-20. doi: 10.1093/intqhc/mzv063

5 Stanhope N, Crowley-Murphy M, Vincent C*, et al.* An evaluation of adverse incident reporting. *J Eval Clin Pract* 1999;5(1):5-12. doi: 10.1046/j.1365-2753.1999.00146.x

6 Vincent C, Amalberti R. Safety in healthcare is a moving target. *BMJ Qual Saf* 2015;24(9):539-40. doi: 10.1136/bmjqs-2015-004403

7 Macrae C, Vincent C. Learning from failure: the need for independent safety investigation in healthcare. *J R Soc Med* 2014;107(11):439-43. doi: 10.1177/0141076814555939

8 Macrae C. The problem with incident reporting. *BMJ Qual Saf* 2016;25(2):71-5. doi: 10.1136/bmjqs-2015-004732

9 Macrae C. Remembering to learn: the overlooked role of remembrance in safety improvement. *BMJ Qual Saf* 2017;26(8):678-82. doi: 10.1136/bmjqs-2016-005547

10 Wrigstad J, Bergström J, Gustafsson P. Mind the gap between recommendation and implementation—principles and lessons in the aftermath of incident investigations: a semi-

For peer review only - http://bmjopen.bmj.com/site/about/guidelines.xhtml

quantitative and qualitative study of factors leading to the successful implementation of recommendations. *BMJ Open* 2014;4 doi: 10.1136/bmjopen-2014-005326
11 Roos af Hjelmsäter E, Ros A, Gäre BA*, et al.* Deficiencies in healthcare prior to suicide and actions to deal with them: a retrospective study of investigations after suicide in Swedish healthcare. *BMJ Open* 2019;9(12):e032290. doi: 10.1136/bmjopen-2019-032290
12 Kellogg KM, Hettinger Z, Shah M*, et al.* Our current approach to root cause analysis: is it contributing to our failure to improve patient safety? *BMJ Qual Saf* 2017;26(5):381-87. doi: 10.1136/bmjqs-2016-005991
13 Mitchell I, Schuster A, Smith K*, et al.* Patient safety incident reporting: a qualitative study of thoughts and perceptions of experts 15 years after 'To Err is Human'. *BMJ Qual Saf* 2016;25(2):92-9. doi: 10.1136/bmjqs-2015-004405
14 Leistikow I, Mulder S, Vesseur J*, et al.* Learning from incidents in healthcare: the journey, not the arrival, matters. *BMJ Qual Saf* 2017;26(3):252-56. doi: 10.1136/bmjqs-2015-004853
15 Shojania KG, Thomas EJ. Trends in adverse events over time: why are we not improving? *BMJ Qual Saf* 2013;22(4):273-77. doi: 10.1136/bmjqs-2013-001935
16 The Swedish Patient Safety Act (SFS 2010:659) [https://www.riksdagen.se/sv/dokument-lagar/dokument/svensk-forfattningssamling/patientsakerhetslag-2010659_sfs-2010-659]. [accessed 2 February 2019].
17 World Health Organization. Suicide data 2018 [Available from: http://www.who.int/mental_health/prevention/suicide/suicideprevent/en/ [accessed 17 October 2018].
18 Mann J, Currier D. Stress, genetics and epigenetic effects on the neurobiology of suicidal behavior and depression. *European Psychiatry* 2010;25(5):268-71.
19 Luoma JB, Martin CE, Pearson JL. Contact with mental health and primary care providers before suicide: a review of the evidence. *Am J Psychiatry* 2002;159(6):909-16. doi: 10.1176/appi.ajp.159.6.909
20 Chock MM, Lin JC, Athyal VP*, et al.* Differences in health care utilization in the year before suicide death: a population-based case-control ctudy. *Mayo Clin Proc* 2019;94(10):1983-93. doi: 10.1016/j.mayocp.2019.04.037
21 Cavanagh JT, Carson AJ, Sharpe M*, et al.* Psychological autopsy studies of suicide: a systematic review. *Psychol Med* 2003;33(3):395-405.
22 Arsenault-Lapierre G, Kim C, Turecki G. Psychiatric diagnoses in 3275 suicides: a meta-analysis. *BMC psychiatry* 2004;4(1):37.
23 Bertolote JM, Fleischmann A. Suicide and psychiatric diagnosis: a worldwide perspective. *World psychiatry* 2002;1(3):181.
24 Kapur N. Health services and suicide prevention. *Journal of Mental Health* 2009;18(1):1-5. doi: 10.1080/09638230802370704
25 Quinlivan L, Littlewood DL, Webb RT*, et al.* Patient safety and suicide prevention in mental health services: time for a new paradigm? *Journal of Mental Health* 2020;29(1):1-5. doi: 10.1080/09638237.2020.1714013
26 Pirkola S, Sund R, Sailas E*, et al.* Community mental-health services and suicide rate in Finland: a nationwide small-area analysis. *The Lancet* 2009;373(9658):147-53.
27 While D, Bickley H, Roscoe A*, et al.* Implementation of mental health service recommendations in England and Wales and suicide rates, 1997–2006: a cross-sectional and before-and-after observational study. *The Lancet* 2012;379(9820):1005-12.
28 Kapur N, Ibrahim S, While D*, et al.* Mental health service changes, organisational factors, and patient suicide in England in 1997–2012: a before-and-after study. *The Lancet Psychiatry* 2016;3(6):526-34.
29 Socialstyrelsens föreskrifter om anmälan av vårdskador enligt lex Maria (SOSFS 2005:28). Stockholm: The Swedish National Board of Health and Welfare.

For peer review only - http://bmjopen.bmj.com/site/about/guidelines.xhtml

30 Socialstyrelsens föreskrifter och allmänna råd om vårdgivares systematiska patientsäkerhetsarbete (HSLF-FS 2017:40). Stockholm: The Swedish National Board of Health and Welfare.

31 Swedish association of local authorities and regions. Risk och händelseanalys [Risk analysis and adverse events analysis]. Stockholm: Swedish association of local authorities and regions 2015.

32 Nelson EC, Batalden PB, Godfrey MM. Quality by design: a clinical microsystems approach. San Fransisco: Jossey-Bass 2007.

33 The national board of health and welfare. Statistikdatabas för dödsorsaker [Statistical databease, cause of death]: The national board of health and welfare; 2020 [Available from: https://sdb.socialstyrelsen.se/if_dor/resultat.aspx [accessed 2020-02-02.

34 Health and Social Care Inspectorate. https://www.ivo.se/publicerat-material/statistik/lex-maria-och-lex-sarah/ [[accessed 2020-02-05.

35 Nicolini D, Waring J, Mengis J. Policy and practice in the use of root cause analysis to investigate clinical adverse events: mind the gap. *Soc Sci Med* 2011;73(2):217-25. doi: 10.1016/j.socscimed.2011.05.010

36 Runeson B, Odeberg J, Pettersson A*, et al.* Instruments for the assessment of suicide risk: a systematic review evaluating the certainty of the evidence. *PLoS one* 2017;12(7):e0180292.

37 Carter G, Milner A, McGill K*, et al.* Predicting suicidal behaviours using clinical instruments: systematic review and meta-analysis of positive predictive values for risk scales. *The British Journal of Psychiatry* 2017;210(6):387-95.

38 Large M, Smith G, Sharma S*, et al.* Systematic review and meta-analysis of the clinical factors associated with the suicide of psychiatric in-patients. *Acta Psychiatrica Scandinavica* 2011;124(1):18-19.

39 Suicide NCIi, Health SiM. The assessment of clinical risk in mental health services: The University of Manchester Manchester, 2018.

40 Berg SH, Rørtveit K, Walby FA*, et al.* Adaptive capacities for safe clinical practice for patients hospitalised during a suicidal crisis: a qualitative study. *BMC psychiatry* 2020;20(1):1-12.

41 Probert-Lindström S, Berge J, Westrin Å*, et al.* Long-term risk factors for suicide in suicide attempters examined at a medical emergency in patient unit: results from a 32-year follow-up study. *BMJ open* 2020;10(10):e038794.

42 Trbovich P, Shojania KG. Root-cause analysis: swatting at mosquitoes versus draining the swamp. *BMJ Qual Saf* 2017;26(5):350-53. doi: 10.1136/bmjqs-2016-006229

43 Cafazzo JA, St-Cyr O. From discovery to design: the evolution of human factors in healthcare. *Healthc Q* 2012;15(April (Special Issue)):24-29.

44 Mills PD, Neily J, Luan D*, et al.* Actions and implementation strategies to reduce suicidal events in the Veterans Health Administration. *Jt Comm J Qual Patient Saf* 2006;32(3):130-41.

45 Percarpio KB, Watts BV, Weeks WB. The effectiveness of root cause analysis: what does the literature tell us? *Jt Comm J Qual Patient Saf* 2008;34(7):391-8. doi: 10.1016/s1553-7250(08)34049-5

46 Macrae C. Close calls: managing risk and resilience in airline flight safety: Springer 2014.

47 Qin P, Nordentoft M. Suicide risk in relation to psychiatric hospitalization: evidence based on longitudinal registers. *Arch Gen Psychiatry* 2005;62(4):427-32. doi: 10.1001/archpsyc.62.4.427

48 Ballard ED PM, Henderson D, et al. Suicide in the medical setting. *Jt Comm J Qual Patient Saf* 2008;34:474-81.

For peer review only - http://bmjopen.bmj.com/site/about/guidelines.xhtml

STROBE 2007 (v4) Statement—Checklist of items that should be included in reports of *cohort studies*

Section/Topic	Item #	Recommendation	Reported on page #
**Title and abstract**	1	(*a*) Indicate the study's design with a commonly used term in the title or the abstract	1
		(*b*) Provide in the abstract an informative and balanced summary of what was done and what was found	2
**Introduction**			
Background/rationale	2	Explain the scientific background and rationale for the investigation being reported	3-4
Objectives	3	State specific objectives, including any prespecified hypotheses	4
**Methods**			
Study design	4	Present key elements of study design early in the paper	4-5
Setting	5	Describe the setting, locations, and relevant dates, including periods of recruitment, exposure, follow-up, and data collection	4
Participants	6	(*a*) Give the eligibility criteria, and the sources and methods of selection of participants. Describe methods of follow-up	4
		(*b*) For matched studies, give matching criteria and number of exposed and unexposed	Not relevant
Variables	7	Clearly define all outcomes, exposures, predictors, potential confounders, and effect modifiers. Give diagnostic criteria, if applicable	4-5
Data sources/ measurement	8*	For each variable of interest, give sources of data and details of methods of assessment (measurement). Describe comparability of assessment methods if there is more than one group	4-5
Bias	9	Describe any efforts to address potential sources of bias	13
Study size	10	Explain how the study size was arrived at	4
Quantitative variables	11	Explain how quantitative variables were handled in the analyses. If applicable, describe which groupings were chosen and why	5
Statistical methods	12	(*a*) Describe all statistical methods, including those used to control for confounding	5
		(*b*) Describe any methods used to examine subgroups and interactions	5
		(*c*) Explain how missing data were addressed	Not relevant
		(*d*) If applicable, explain how loss to follow-up was addressed	Not relevant
		(*e*) Describe any sensitivity analyses	Not relevant
**Results**			

For peer review only

Participants	13*	(a) Report numbers of individuals at each stage of study—eg numbers potentially eligible, examined for eligibility, confirmed eligible, included in the study, completing follow-up, and analysed	6
		(b) Give reasons for non-participation at each stage	Not relevant
		(c) Consider use of a flow diagram	Not relevant
Descriptive data	14*	(a) Give characteristics of study participants (eg demographic, clinical, social) and information on exposures and potential confounders	6-7
		(b) Indicate number of participants with missing data for each variable of interest	Not relevant
		(c) Summarise follow-up time (eg, average and total amount)	Not relevant
Outcome data	15*	Report numbers of outcome events or summary measures over time	7-10
Main results	16	(*a*) Give unadjusted estimates and, if applicable, confounder-adjusted estimates and their precision (eg, 95% confidence interval). Make clear which confounders were adjusted for and why they were included	7-10
		(*b*) Report category boundaries when continuous variables were categorized	Not relevant
		(*c*) If relevant, consider translating estimates of relative risk into absolute risk for a meaningful time period	Not relevant
Other analyses	17	Report other analyses done—eg analyses of subgroups and interactions, and sensitivity analyses	Not relevant
**Discussion**			
Key results	18	Summarise key results with reference to study objectives	13
**Limitations**			
Interpretation	20	Give a cautious overall interpretation of results considering objectives, limitations, multiplicity of analyses, results from similar studies, and other relevant evidence	11-13
Generalisability	21	Discuss the generalisability (external validity) of the study results	11-13
**Other information**			
Funding	22	Give the source of funding and the role of the funders for the present study and, if applicable, for the original study on which the present article is based	14

*Give information separately for cases and controls in case-control studies and, if applicable, for exposed and unexposed groups in cohort and cross-sectional studies.

Note: An Explanation and Elaboration article discusses each checklist item and gives methodological background and published examples of transparent reporting. The STROBE checklist is best used in conjunction with this article (freely available on the Web sites of PLoS Medicine at http://www.plosmedicine.org/, Annals of Internal Medicine at http://www.annals.org/, and Epidemiology at http://www.epidem.com/). Information on the STROBE Initiative is available at www.strobe-statement.org.

For peer review only
